# A compound memristive synapse model for statistical learning through STDP in spiking neural networks

**DOI:** 10.3389/fnins.2014.00412

**Published:** 2014-12-16

**Authors:** Johannes Bill, Robert Legenstein

**Affiliations:** Faculty of Computer Science and Biomedical Engineering, Institute for Theoretical Computer Science, University of TechnologyGraz, Austria

**Keywords:** neuromorphic, synapse, synaptic plasticity, STDP, memristor, WTA, Bayesian inference, unsupervised learning

## Abstract

Memristors have recently emerged as promising circuit elements to mimic the function of biological synapses in neuromorphic computing. The fabrication of reliable nanoscale memristive synapses, that feature continuous conductance changes based on the timing of pre- and postsynaptic spikes, has however turned out to be challenging. In this article, we propose an alternative approach, the compound memristive synapse, that circumvents this problem by the use of memristors with binary memristive states. A compound memristive synapse employs multiple bistable memristors in parallel to jointly form one synapse, thereby providing a spectrum of synaptic efficacies. We investigate the computational implications of synaptic plasticity in the compound synapse by integrating the recently observed phenomenon of stochastic filament formation into an abstract model of stochastic switching. Using this abstract model, we first show how standard pulsing schemes give rise to spike-timing dependent plasticity (STDP) with a stabilizing weight dependence in compound synapses. In a next step, we study unsupervised learning with compound synapses in networks of spiking neurons organized in a winner-take-all architecture. Our theoretical analysis reveals that compound-synapse STDP implements generalized Expectation-Maximization in the spiking network. Specifically, the emergent synapse configuration represents the most salient features of the input distribution in a Mixture-of-Gaussians generative model. Furthermore, the network's spike response to spiking input streams approximates a well-defined Bayesian posterior distribution. We show in computer simulations how such networks learn to represent high-dimensional distributions over images of handwritten digits with high fidelity even in presence of substantial device variations and under severe noise conditions. Therefore, the compound memristive synapse may provide a synaptic design principle for future neuromorphic architectures.

## 1. Introduction

A characteristic property of massively parallel computation in biological and artificial neural circuits is the need for intensive communication between neuronal elements. As a consequence, area- and energy consumption of neuromorphic circuits is often dominated by those circuits that implement synaptic transmission between neural elements (Schemmel et al., 2008). Biological synapses are highly dynamic computational entities, exhibiting plasticity on various time scales (Malenka and Bear, 2004). In order to capture the most salient of these aspects, silicon synapses thus demand extensive circuitry if implemented in CMOS technology. On this account, novel nanoscale circuit elements have recently gained interest in the field of neuromorphic engineering as a promising alternative solution for the implementation of artificial synaptic connections. In particular, for mixed-signal neuromorphic CMOS architectures, which combine traditional digital circuits with analog components, memristors are considered a promising class of circuit elements due to high integration densities, synapse-like plasticity dynamics, and low power consumption (Choi et al., 2009; Jo et al., 2010; Kuzum et al., 2011; Indiveri et al., 2013). One particularly important feature of memristors is that their electrical resistance (often termed “memristance”) can be altered in a persistent manner by applying a voltage to its terminals, leading to the eponymous perception of memristors as resistors with memory. As an important application of this property, it was shown that the memristance can be changed based on the spike timings of the pre- and postsynaptic neurons in a manner that approximates spike-timing dependent plasticity (STDP), a plasticity rule that is believed to represent a first approximation for the changes of synaptic efficacies in biological synapses (Markram et al., 1997; Caporale and Dan, 2008; Markram et al., 2012). On the level of single synapses, this important property has been confirmed experimentally in real memristors (Mayr et al., 2012) and has been included into computational models of memristive plasticity (Serrano-Gotarredona et al., 2013). On the network level, models of memristive STDP were employed in computer simulations to demonstrate the potential applicability of neuromorphic designs with memristive synapses to pattern recognition tasks (Querlioz et al., 2011).

In practice however, the production of functional memristive synapses with nanoscale dimensions has proven difficult, mainly due to large device variations and their unreliable behavior. It turned out to be particularly challenging to fabricate reliable nanoscale memristive synapses that feature a continuous spectrum of conductance values. As an alternative solution, it was proposed to employ bistable memristors as neuromorphic synapses instead since they exhibit a high degree of uniformity (Fang et al., 2011) and high durability (Jo et al., 2009b). For switching between their two stable conductance states, bistable memristors can be operated in a deterministic as well as in a stochastic regime (Jo et al., 2009b).

Using machine learning theory, we show in this article that stochastically switching bistable memristors become computationally particularly powerful in mixed-signal neuromorphic architectures when multiple memristors are combined to jointly form one synapse. Such a joint operation of multiple memristors can be interpreted as the collective function of ion channels in biological synapses (Indiveri et al., 2013). Concretely, we propose the *compound memristive synapse model* which employs *M* bistable memristors operating in parallel to form a single synaptic weight between two neurons. To implement synaptic plasticity, we employ standard STDP pulsing schemes (Querlioz et al., 2011; Serrano-Gotarredona et al., 2013) and exploit the stochastic nature of memristive switching (Jo et al., 2009b; Gaba et al., 2013; Suri et al., 2013; Yu et al., 2013). For the analysis of the resulting plasticity dynamics, we perceive individual memristors as binary stochastic switches. This abstract description was previously utilized to capture the most salient features of experimentally observed memristive switching (Suri et al., 2013) and appears compatible with pivotal aspects of the experimental literature (Jo et al., 2009b; Gaba et al., 2013).

We show analytically and through computer simulations that the change of the synaptic efficacy for a given pairing of pre- and postsynaptic spikes follows an STDP-like plasticity rule such that the expected weight change depends on the momentary synaptic weight in a stabilizing manner. The resulting *compound-synapse STDP* enables a synapse to attain many memristive states depending on the history of pre- and postsynaptic activity. A stabilizing weight dependence of synaptic plasticity exists in biological synapses (Bi and Poo, 1998) and has been shown to facilitate learning and adaptation in neural systems (Van Rossum et al., 2000; Morrison et al., 2007). In particular, it has been shown in Nessler et al. (2013) that in stochastic winner-take-all (WTA) architectures, STDP with stabilizing weight dependence implements an online Expectation-Maximization algorithm. When exposed to input examples, neurons in the WTA network learn to represent the hidden causes of the observed input in a well-defined generative model. This adaptation proceeds in a purely unsupervised manner. We adopt a similar strategy here and apply the compound memristive synapse model in a network of stochastically spiking neurons arranged in a WTA architecture. We show analytically that compound-synapse STDP optimizes the synaptic efficacies such that the WTA network neurons in the hidden layer represent the most salient features of the input distribution in a Mixture-of-Gaussians generative model. After training, the network performs Bayesian inference over the hidden causes for the given input pattern. We show in computer simulations that such networks are able to learn to represent high-dimensional distributions over images of handwritten digits. After unsupervised training, the network transforms noisy input spike-patterns into a sparse and reliable spike code that supports classification of images. It turns out that even small compound synapses, consisting of only four bistable constituents per synapse, are sufficient for reliable image classification in our simulations. We furthermore show that the proposed model is able to represent the input distribution with high fidelity even in the presence of substantial device variations and under severe noise conditions. These findings render the compound synapse model a promising design principle for novel high-density, low-power mixed-signal CMOS architectures.

## 2. Results

### 2.1. Stochastic memristors as plastic synapses

**Figure 1:**
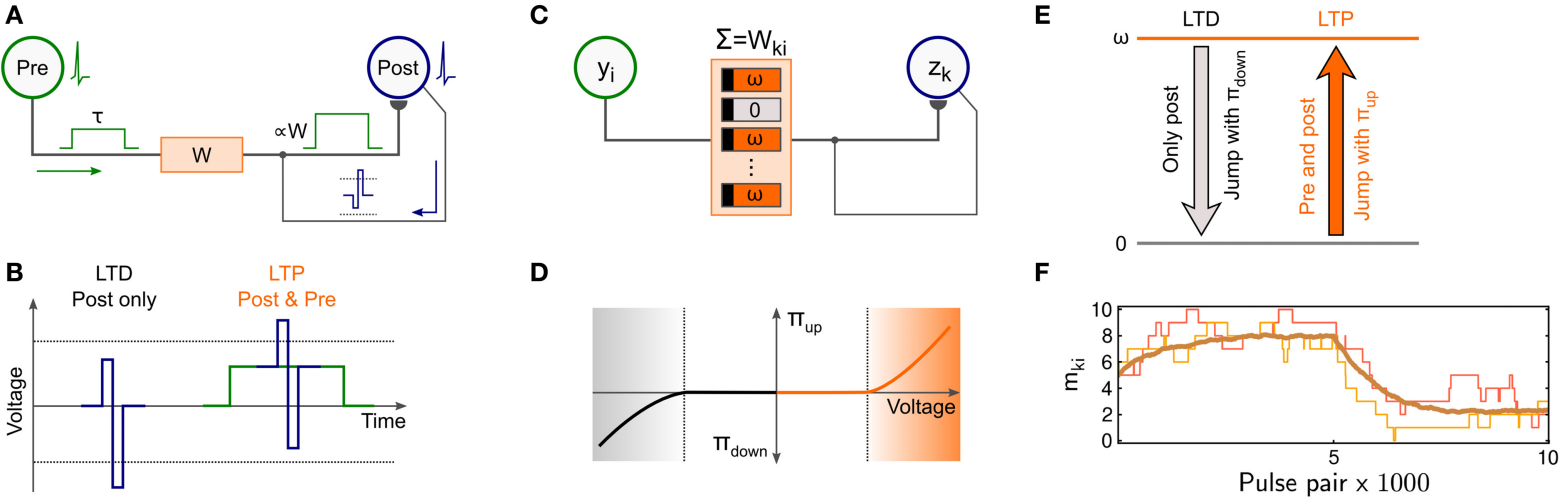
**Compound memristive synapse model with stochastic memristors**. **(A)** STDP pulsing scheme. Input spikes elicit a rectangular voltage trace (green, left) that is sent to the presynaptic terminal of the memristor synapse. Post-synaptic spikes elicit a brief voltage pulse (blue) which is sent back to the synapse. **(B)** Solitary postsynaptic spikes trigger LTD since the voltage exceeds the lower threshold of the memristor. Simultaneous pre- and postsynaptic spikes trigger LTP since the voltage exceeds the upper threshold. **(C)** Compound memristive synapse model. A synapse is composed of *M* bistable memristors operating in parallel. Each memristor can either be active (weight ω) or inactive (weight 0). The total synaptic weight *W*_*ki*_ between input neuron *Y*_*i*_ and network neuron *z*_*k*_ is the sum of the individual memristor weights. **(D)** Bistable memristors switch stochastically between the active and inactive state depending on the applied voltage difference across its terminals. Switching to the active state (inactive state) occurs with probability π_up_ (π_down_) if a certain threshold voltage (dotted line) is exceeded. **(E)** Summary of stochastic transitions for compound-synapse STDP. **(F)** In an STDP pairing experiment, the stabilizing weight dependence of compound synapse plasticity governs convergence to a dynamic equilibrium. 10,000 plasticity pulses were applied to a synapse with *M* = 10 constituents. During the first half, 80% (20%) of the events were of LTP (LTD) type. During the second half, the probability for LTP (LTD) events was inverted to 20% (80%). Thin lines: number of active memristors *m*_*ki*_(*t*) for two example simulation runs. Thick line: Average 〈*m*_*ki*_(*t*)〉 over 100 runs. The average weight converges to a dynamic equilibrium.

Memristors have gained increasing attention in neuromorphic engineering as possible substrates for plastic synapses (Jo et al., 2010) due to the possibility to change their electrical conductance without the requirement of extensive supporting circuitry. Recently, Zamarreño-Ramos et al. (2011) and Querlioz et al. (2011) have proposed a pulsing scheme to realize spike-timing dependent plasticity (STDP) with memristive synapses in response to pre- and postsynaptic activity as sketched in Figure 1A: Pre-synaptic spikes trigger a rectangular voltage pulse of duration τ (shown in green) that is sent to the memristor's presynaptic terminal. Similarly, postsynaptic neurons send back a copy of their spikes to the postsynaptic terminal as a brief voltage pulse (shown in blue). The combined effect of these pulses was shown to trigger STDP-type plasticity in the memristor as illustrated in Figure 1B, where we adopted the convention to measure the voltage drop at the memristor as “presynaptic minus postsynaptic potential.” If only the postsynaptic neuron spikes (Figure 1B, left), the voltage pulse exceeds the lower threshold of the memristor, leading to long-term depression (LTD). Conversely, if a postsynaptic spike follows a presynaptic spike within duration τ (Figure 1B, right), the combined voltage trace exceeds the memristor's upper threshold, thus resulting in long-term potentiation (LTP). Pre-synaptic pulses alone do not trigger any plasticity. Overall, the memristor's conductance obeys an STDP-type plasticity rule.

The direct implementation of the above plasticity rule in mixed-signal neuromorphic architectures, however, faces practical challenges due to the continuous spectrum of the conductance values it relies on. Memristors that support a (quasi-) continuous spectrum of memristive states often suffer from instabilities to maintain their conductance value (“volatility”) and typically show unreliable changes under repetitive application of identical pulses.

Here we explore an alternative approach that employs multiple bistable memristors, that support only two distinct conductance states per memristor, to jointly form one synapse. Such devices were reported to exhibit a high degree of uniformity (Fang et al., 2011). Concretely, we propose a synapse model which employs *M* bistable memristors operating in parallel to form a single synaptic weight *W*_*ki*_ between the i-th input neuron *y*_*i*_ and the k-th network neuron *z*_*k*_. The model which we refer to as *compound memristive synapse* is sketched in Figure 1C. Each memristor is assumed to provide two stable states: a high-conductive (active) state and a low-conductive (inactive) state. Since the dynamic range of memristors typically covers several orders of magnitude, the weight contribution of inactive memristors is almost negligible. In line with this notion, each inactive memristor contributes weight 0 in the synapse model and each active memristor contributes weight ω. Since parallel conductances sum up, the total weight of the compound memristive synapse reads

(1) $$W_{ki}=\omega \cdot m_{ki}$$

with *m*_*ki*_ ∈ {0, 1, …, *M*} denoting the number of active memristors. As a consequence, a compound memristive synapse supports *M* + 1 discrete weight levels, ranging from 0 to the maximum weight *W*_max_ = ω · *M*.

Plasticity in this synapse model naturally emerges from transitions between the active and inactive state of the bistable constituents. However, deterministic transitions, which are, for instance, desirable in memristor-based memory cells, impair the performance in a neuromorphic online learning setup with compound memristive synapses: If all constituents of a synapse, that experience the same pre- and postsynaptic spikes, change their state simultaneously, the compound weight toggles between *W*_*ki*_ = 0 and *W*_*ki*_ = *W*_max_ depending on the latest pulse pair, not showing any gradual trace of memory formation as required for STDP.

A possible remedy to this issue can be found in memristors that exhibit stochastic rather than deterministic switching between their stable states. Yu et al. (2013), for instance, reported stochastic transitions in HfO_x_/TiO_x_ memristors (from the class of anion-based memristors) and explored in computer simulations how stochastic bistable memristors could be used in a neuromorphic learning architecture. Similar studies explored the usability of stochastically switching bistable cation-based memristive materials (Jo et al., 2009b; Gaba et al., 2013; Suri et al., 2013). In these nanoscale devices, changes in the memristance were shown to be dominated by the formation of a single conductive filament (Jo et al., 2009b). Stochastic switching was demonstrated for both directions (active ↔ inactive) (Suri et al., 2013) with switching probabilities being adjustable via the duration (Gaba et al., 2013; Suri et al., 2013) and amplitude (Jo et al., 2009b; Gaba et al., 2013) of the voltage applied across the terminals. These observations have led to the conclusion that “switching can be fully stochastic” with switching probabilities being almost unaffected by the rate at which consecutive plasticity pulses are delivered (Gaba et al., 2013). Furthermore, bistable memristors can be extremely durable, not showing any notable degradation over hundreds of thousands of programming cycles (Jo et al., 2009b). Owing to these pivotal properties, Suri et al. (2013) proposed an STDP-type plasticity rule that perceives bistable memristors as simple stochastic switches.

Here we generalize this idea to compound memristive synapses and investigate the computational function of the arising plasticity rules in spiking networks from a machine learning perspective. Figure 1D illustrates a simple model of stochastic switching of individual memristors that employs the STDP-pulsing scheme from Zamarreño-Ramos et al. (2011) and Querlioz et al. (2011) discussed above: If the voltage between the pre- and postsynaptic terminal of a device exceeds a certain threshold (dotted lines) for the duration of the back-propagating spike signal, stochastic switching may occur. Inactive memristors jump to the active state with probability π_up_ provided a sufficiently strong positive voltage, resulting in stochastic LTP. Similarly, active memristors turn inactive with probability π_down_ given a sufficiently strong negative voltage, leading to stochastic LTD. No switching occurs if only a small (or zero) voltage is applied, or if the memristor is already in the respective target state. Since the applied pre- and postsynaptic pulse amplitudes are free parameters in the model, the jumping probabilities π_up_ and π_down_ can be controlled, to a certain extent, by the experimenter. This simple model captures the most salient aspects of stochastic switching in bistable memristive devices as discussed above, cp. also Suri et al. (2013) and the Discussion section. In order to distinguish the abstract memristor model from physical memristive devices, we will in the following refer to the model memristors as “stochastic switches,” “constituents,” or simply as “switches” for the sake of brevity. Furthermore, we refer to the resulting stochastic plastic behavior of compound memristive synapses in response to pre-post spike pairs, summarized in Figure 1E, as *compound-synapse STDP*.

From the transition probabilities of individual stochastic switches we can calculate the expected temporal weight change $\langle \frac{d}{dt}W_{ki}\rangle$ of the compound memristive synapse as a function of pre- and postsynaptic activity. Formally, we denote the presence of a rectangular input pulse (green in Figure 1A) of the i-th input by *y*_*i*_(*t*) = 1 (and the absence by *y*_*i*_(*t*) = 0). The brief pulses that are sent back from a postsynaptic neuron *z*_*k*_ to the synapse (blue in Figure 1A) are formally treated as point events at the spike times of the postsynaptic neuron. We denote the spike time of the *f*^th^ spike of neuron *z*_*k*_ by *t*^*f*^_*k*_. The spike train *s*_*k*_(*t*) of a neuron *z*_*k*_ is formally defined as the sum of Dirac delta pulses δ(·) at the spike times: *s*_*k*_(*t*) = ∑_*f*_ δ(*t* − *t*^*f*^_*k*_). When a synaptic efficacy *W*_*ki*_ is subject to a stochastic LTP update, there are (*M* − *m*_*ki*_) constituents in the compound memristive synapse that are currently inactive and could undergo an LTP transition. Each constituent independently switches to its active state with probability π_up_, thereby contributing ω to the compound weight *W*_*ki*_. Hence, the expected weight change for the LTP condition reads (*M* − *m*_*ki*_) ω π_up_. A similar argumentation applies to the LTD case. In summary, considering that plasticity always requires a postsynaptic spike, and that LTP is induced in the presence of a presynaptic pulse (*y*_*i*_(*t*) = 1), while LTD is induced in the absence of a presynaptic pulse (*y*_*i*_(*t*) = 0), the expected weight change of the compound memristive synapse reads:

(2) $$\begin{array}{l} \langle \frac{d}{dt}W_{ki}\rangle =s_{k}(t)\cdot [\underset{\text{LTP}}{\underset{︸}{\left(M-m_{ki})\omega \pi_{\text{up}}y_{i}(t\right)}}\\ \;-\underset{\text{LTD}}{\underset{︸}{m_{ki}\omega \pi_{\text{down}}(1-y_{i}(t))}}]\\ \;=s_{k}(t)\cdot [M\omega \pi_{\text{up}}y_{i}(t)-m_{ki}\omega \pi_{\text{up}}y_{i}(t)\\ \;-m_{ki}\omega \pi_{\text{down}}+m_{ki}\omega \pi_{\text{down}}y_{i}(t)]\\ \;=s_{k}(t)\cdot \left[W_{\text{max}}\pi_{\text{up}}y_{i}(t)-W_{ki}\pi_{\text{down}}\right]\\ \;+s_{k}(t)W_{ki}y_{i}(t)\cdot (\pi_{\text{down}}-\pi_{\text{up}})\;. \end{array}$$

In order to obtain a simple closed form solution of the weight changes, we set π_up_ = π_down_, i.e., the probability of potentiation of a single switch under LTP equals its probability of depression under LTD. This choice will facilitate the theoretical analysis of learning in a spiking network, later on. In a hardware implementation, the switching probabilities could be adjusted via the pre- and postsynaptic amplitudes of the STDP pulses. We obtain the following closed form solution for the expected weight change:

(3) $$\langle \frac{d}{dt}W_{ki}\rangle =\pi_{\text{up}}W_{\text{max}}\cdot s_{k}(t)\cdot \left[y_{i}(t)-W_{ki}/W_{\text{max}}\right]\;.$$

Notably, the plasticity rule (3) differs from standard additive STDP rules in that it includes the weight dependent term *W*_*ki*_/*W*_max_. This weight dependence has its origin in the varying number of (in-)active stochastic switches *m*_*ki*_ that could actually undergo plastic changes and is in line with a prominent finding from neurobiology (Bi and Poo, 1998): Relative changes Δ*W*_*ki*_/*W*_*ki*_ become weaker for strong weights under LTP, while under LTD the relative change is weight-independent. Studies in computational neuroscience, see e.g., Van Rossum et al. (2000), found that this type of weight dependence facilitates the formation of stable connections in spiking networks.

In Figure 1F, we illustrate the stochastic convergence of a compound synaptic weight to a stable, dynamic equilibrium in a simple STDP pairing experiment. The synapse consists of *M* = 10 bistable switches with switching probabilities set to π_up_ = π_down_ = 0.001. These synapse parameters will also be used in network level simulations, later on. In a small computer simulation, 5 of the 10 constituents are initially set active (*m*_*ki*_(*t* = 0) = 5). Then, 5000 postsynaptic spikes are sent to the postsynaptic terminal of the synapse for triggering stochastic switching in the bistable constituents. 80% of these events are randomly paired with a presynaptic pulse, i.e., 80% of the events are of LTP type and 20% are of LTD type. After 5000 plasticity pulses, the statistics are inverted to 20% LTP and 80% LTD events for another 5000 plasticity pulses. The thin lines in Figure 1F show the evolution of *m*_*ki*_(*t*) in this STDP pairing experiment for two independent simulation runs. In the first half, the synaptic weight tends to settle around higher weight values, while in the second half, it stochastically declines toward lower weight values. The gradual convergence of the average weight, as suggested by Equation (3), becomes apparent by taking the mean over 100 simulation runs (thick line). The stabilizing weight dependence of the STDP rule leads to convergence to a dynamic equilibrium such that the mean value shows slow, continuous changes as expected from the theory. Individual synapses fluctuate stochastically around this mean.

Equation (3) is reminiscent of a theoretically derived synaptic plasticity rule for statistical model optimization in spiking neural networks proposed by Nessler et al. (2013). Building upon the theoretical approach developed by Nessler et al. (2013), we will next turn to the question how to conceive stochastic learning with compound memristive synapses from a Bayesian perspective as unsupervised model optimization via a powerful optimization method that is known as Expectation-Maximization in the machine learning literature.

### 2.2. Compound memristive synapses in winner-take-all networks

The winner-take-all (WTA) network structure is a ubiquitous circuit motif in neocortex (Douglas and Martin, 2004; Lansner, 2009) and is often utilized in neuromorphic engineering (Mead and Ismail, 1989; Indiveri, 2000). Recently, WTA networks attracted increasing attention in theoretical studies on statistical learning (Habenschuss et al., 2012; Keck et al., 2012; Habenschuss et al., 2013; Nessler et al., 2013; Kappel et al., 2014) because their comparatively simple network dynamics facilitate a comprehensive mathematical treatment. In this section, we introduce the WTA architecture that we will use to study the learning capabilities of spiking neural networks with compound memristive synapses subject to STDP.

**Figure 2:**
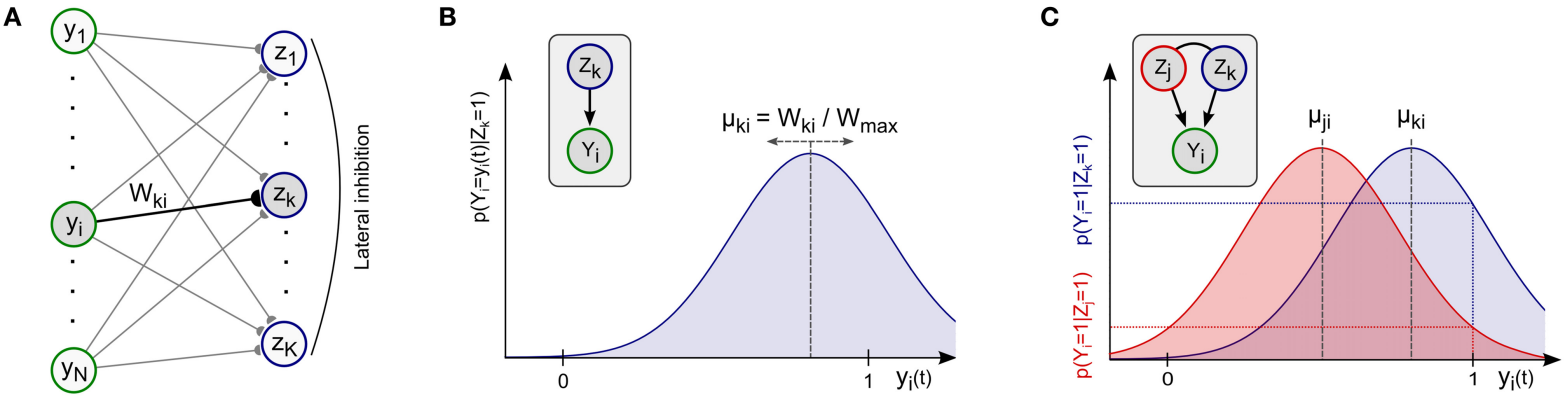
**Spiking network for probabilistic inference and online learning**. **(A)** Winner-take-all network architecture with lateral inhibition and synaptic weights *W*_*ki*_. **(B)** Network neurons *z*_*k*_ implicitly maintain a Gaussian likelihood function for each input *Y*_*i*_ in their afferent synaptic weights *W*_*ki*_. The mean μ_*ki*_ of the distribution is encoded by the fraction *W*_*ki*_/*W*_max_ = *m*_*ki*_/*M* of active switches in the compound memristive synapse, i.e., stronger synaptic weights *W*_*ki*_ correspond to higher mean values μ_*ki*_. Inset: Local implicit graphical model. **(C)** Illustration of Bayesian inference for two competing network neurons *z*_*k*_, *z*_*j*_ and one active input *Y*_*i*_(*t*) = 1. Different means μ_*ki*_, μ_*ji*_ encoded in the weights give rise to different values in the likelihood function and shape the posterior distribution according to Bayes rule.

The WTA network architecture is sketched in Figure 2A. The network consists of *N* spiking input neurons *y*_1_, …, *y*_*N*_ and *K* spiking network neurons *z*_1_, …, *z*_*K*_ with all-to-all connectivity in the forward synapses. Lateral inhibition introduces competition among the network neurons. Network neurons are stochastic spike response neurons (Gerstner and Kistler, 2002) with the membrane potential *u*_*k*_ of network neuron *z*_*k*_ being given by

(4) $$u_{k}(t)=b_{k}+\sum_{i=1}^{N}W_{ki}\cdot y_{i}(t)\;.$$

The membrane potential *u*_*k*_(*t*) integrates the inputs *y*_*i*_(*t*), i.e., the rectangular voltage pulses following each input spike, linearly through the synaptic weights *W*_*ki*_. The parameter *b*_*k*_ denotes the intrinsic excitability of the neuron and controls its general disposition to fire. In Methods we outline how the linear membrane potential (4) can be realized with leaky integrators, a common neuron model in neuromorphic designs. For the spike response of the network neurons *z*_*k*_, a stochastic firing mechanism is employed. In the WTA network, neurons *z*_*k*_ spike in a Poissonian manner with instantaneous firing rate ρ_*k*_(*t*) that depends on the membrane potential *u*_*k*_(*t*) and on lateral inhibition *u*_inh_(*t*):

(5) $$\rho_{k}(t)=r_{\text{net}}\cdot e^{u_{k}(t)-u_{\text{inh}}(t)}\;,$$

with a constant *r*_net_ > 0 that scales the overall firing rate of the network. In other words, the neuron spikes with probability ρ_*k*_(*t*) · δ*t* in a small time window δ*t*→ 0. The inhibitory contribution $u_{\text{inh}}(t):=\log\sum_{j\;=\;1}^{K}\exp(u_{j}(t))$ summarizes the effect of lateral inhibition in the network and introduces WTA-competition between the network neurons to fire in response to a given stimulus *y*_1_(*t*), …, *y*_*N*_(*t*). Notably, the exponential relationship (5) between an idealized membrane potential and neuronal firing is consistent with biological findings about the response properties of neocortical pyramidal neurons (Jolivet et al., 2006).

The feed-forward synapses from inputs *y*_*i*_ to network neurons *z*_*k*_ are implemented as compound memristive synapses and their synaptic weights *W*_*ki*_ are adapted through stochastic STDP as described above. The intrinsic excitabilities *b*_*k*_ are adapted according to a homeostatic plasticity rule (Habenschuss et al., 2012) that ensures that all network neurons take part in the network response and thus facilitates the emergence of a rich neural representation that covers the entire input space. Besides its observed stabilizing effect (Querlioz et al., 2011), homeostatic intrinsic plasticity plays a distinct computational role when combined with synaptic learning: Network neurons that maintain many strong synapses *W*_*ki*_ gain an “unrightful advantage” during WTA competition over neurons that are specialized on low-activity input patterns (and therefore maintain weaker weights). A detailed analysis of the learning dynamics in the network shows that, in order to compensate for this advantage, the former must be burdened with a lower excitability *b*_*k*_ than the latter. A formal definition of the homeostatic plasticity mechanism and a discussion of its computational role from a theory perspective are provided in Methods, see also Habenschuss et al. (2012).

### 2.3. Memristive synapses support inference and online learning

In this section, we thoroughly analyze the learning effects of STDP in compound memristive synapses in the stochastic WTA network model. For the mathematical analysis, we describe the inputs *Y*_*i*_(*t*) and the stochastic neuron responses *s*_*k*_(*t*) with the help of probability theory. To this end, we perceive the spiking activity of the input neurons *y*_*i*_ and network neurons *z*_*k*_ as samples of random variables (RVs) *Y*_*i*_ and *Z*_*k*_ respectively. Consistent with the assumption that spikes from input neurons produce voltage pulses of duration τ in the circuit implementation (see Figure 1A), we set *Y*_*i*_ = *y*_*i*_(*t*), i.e., *Y*_*i*_ = 1 if input neuron *y*_*i*_ spiked within [*t* − τ, *t*] and *Y*_*i*_ = 0 otherwise. The assignment of output spikes *s*_*k*_(*t*) to RVs *Z*_*k*_ is different: The random variable *Z*_*k*_ labels the winner of the WTA network at spike times of network neurons. Hence, the value of *Z*_*k*_ is only defined at the moments when one of the *K* network neurons spikes, i.e., when the spike train *s*_*j*_(*t*) ≠ 0 for some neuron *z*_*j*_. In this case, *Z*_*k*_ encodes which neuron spiked, and we set *Z*_*k*_ = 1 if *k* = *j* and *Z*_*k*_ = 0 if *k* ≠ *j*.

Using this interpretation of neural activity as realizations of RVs, the network's stochastic response *s*_*k*_(*t*) to an input configuration ***y***(*t*) = (*y*_1_(*t*), …, *y*_*N*_(*t*)) gives rise to a conditional probability distribution *p*_net_(***Z*** | ***Y***) over the network RVs ***Z***: = (*Z*_1_, …, *Z*_*K*_) conditioned on the input RVs ***Y***: = (*Y*_1_, …, *Y*_*N*_). In line with the definition of the RVs *Z*_*k*_, the distribution *p*_net_(**Z** | ***Y***) describes the network response only when one of the network neurons *z*_*k*_ fires. The response distribution *p*_net_(**Z** | ***Y*** = ***y***(*t*)) for any fixed input configuration ***y***(*t*) can directly be calculated from Equations (4) and (5) (note that the probability *p*_net_(*Z*_*k*_ = 1 | ***Y*** = ***y***(*t*)) for an individual RV *Z*_*k*_ to be active is proportional to the firing rate ρ_*k*_(*t*) of neuron *z*_*k*_):

(6) $$\begin{array}{l} p_{\text{net}}(Z_{k}=1|Y=y(t))=\rho_{k}(t)/r_{\text{net}}=e^{u_{k}(t)-u_{\text{inh}}(t)}\\ \;=\frac{e^{b_{k}+\sum_{i=1}^{N}W_{ki}\cdot y_{i}(t)}}{\sum_{j=1}^{K}e^{b_{j}+\sum_{i=1}^{N}W_{ji}\cdot y_{i}(t)}}\;. \end{array}$$

Equation (6) fully characterizes the network response distribution *p*_*net*_(***Z*** | ***Y***) for any given input ***Y*** = ***y***(*t*). We next turn to the question how the response distribution *p*_*net*_(***Z*** | ***Y***) can be understood as the result of a meaningful probabilistic computation. Specifically, we will show that the spike response of the WTA network approximates the Bayesian posterior distribution during inference in a well-defined probabilistic model. This probabilistic model is implicitly encoded in the synaptic weights *W*_*ki*_, and synaptic plasticity can thus be perceived as an ongoing refinement of the involved probability distributions. Indeed, the STDP rule (3) of the compound memristive synapses turns out to be optimal in the sense that it instantiates *generalized Expectation-Maximization* in the WTA network, a powerful algorithm for unsupervised learning from machine learning theory. Our findings build upon theoretical work on synaptic learning in spiking neural networks from Nessler et al. (2009, 2013) and Habenschuss et al. (2012, 2013).

The key idea for identifying the response distribution *p*_net_(***Z*** | ***Y***) as the result of a Bayesian computation, is to hypothetically reverse the network computation and view the spike response of a network neuron *z*_*k*_ as the *hidden cause* behind the observed input ***y***(*t*). In this view, the network is treated as a *generative model* that implicitly defines a prior distribution *p*(***Z***) over hidden causes *Z*_*k*_ and a set of likelihood distributions *p*(***Y*** | *Z*_*k*_ = 1), one for each hidden cause *Z*_*k*_. The shape of the distributions is defined by the parameters of the network, e.g., the synaptic weights *W*_*ki*_. An important property of these implicitly encoded distributions–that also motivates the term “generative model”–is that they give rise to a (hypothetical) distribution over the inputs, $p(Y=Y(t))=\sum_{k\;=\;1}^{K}p(Y=Y(t)|Z_{k}=1)\;\cdot \;p(Z_{k}=1)$. This equation also explains why a RV *Z*_*k*_ is called a hidden cause: If we observe an input vector ***y***(*t*), that has high probability only in one of the likelihood distributions *p*(***Y*** = ***y***(*t*) | *Z*_*k*_ = 1), then we can consider the RV *Z*_*k*_ as a likely (but unobservable) cause for the observation according to the generative model. A common objective in machine learning theory, known as *Maximum-Likelihood learning*, is to find parameters that bring the implicit distribution *p*(***Y***) of the generative model as close as possible to the distribution of the actually observed input. Then the hidden causes of the generative model are expected to represent important features of the observed input (e.g., some typical input clusters). Leaving the hypothetical generative perspective again, in the network's real operation an input ***y***(*t*) is presented to the network and the hidden causes *Z*_*k*_ need to be inferred (e.g., the cluster the input ***y***(*t*) belongs to). The mathematically correct result of this inference is given by Bayes rule

(7) $$p(Z_{k}=1|Y=y(t))\propto p(Z_{k}=1)\cdot p(Y=y(t)|Z_{k}=1)$$

which combines the likelihood *p*(***Y*** = ***y***(*t*) | *Z*_*k*_ = 1) with the prior *p*(*Z*_*k*_ = 1).

Nessler et al. (2013) showed that in a WTA network architecture, that evolves according to Equations (4) and (5), the synaptic weights *W*_*ki*_ can be understood as an implicit neural encoding of likelihood distributions *p*(***Y*** | *Z*_*k*_ = 1), and that the network response *p*_net_(***Z*** | ***Y***) approximates the posterior distribution according to Equation (7). Hence, WTA networks can be regarded as implicit generative models. Furthermore–and even more importantly from a theoretical perspective–Nessler et al. (2013) showed that the implicit likelihood model, that is encoded in the weights *W*_*ki*_, can be optimized in an unsupervised manner by a weight-dependent STDP rule. Indeed, there exists a tight link between the type of weight dependence in the STDP rule and the type of implicit likelihood model it optimizes. The exponential weight dependence in Nessler's rule, however, differs from the linear weight dependence we identified for the plasticity rule in Equation (3). This raises the question what type of implicit likelihood model is encoded and optimized by the compound memristive synapses.

An intuition about an appropriate probabilistic interpretation of compound-synapse STDP can be obtained from the equilibrium points of the plasticity rule (3). We first observe, that plasticity is always triggered by a postsynaptic spike, i.e., *Z*_*k*_ = 1 for some neuron *z*_*k*_. In the spirit of spike-triggered averaging (Simoncelli et al., 2004), we can then study the conditional distribution *p*(*Y*_*i*_ = *y*_*i*_(*t*) | *Z*_*k*_ = 1) of an input *Y*_*i*_ at the moment of the network response since the coincidence of pre- and postsynaptic spiking activity is the driving force behind any weight change in the STDP rule. By assuming that plasticity has converged to a dynamic equilibrium, the average contributions of LTP and LTD cancel each other, i.e., ${\langle \frac{d}{dt}W_{ki}\rangle }_{p(Y_{i}|Z_{k}=1)}=0$. From this condition, we obtain the following relation between the synaptic weight *W*_*ki*_ and the conditional input distribution *p*(*Y*_*i*_ | *Z*_*k*_ = 1):

(8) $$\begin{array}{l} \;0\overset{!}{=}\langle \frac{d}{dt}W_{ki}\rangle p(Y_{i}|Z_{k}=1)\\ \;={\langle \pi_{\text{up}}W_{\text{max}}\cdot \left[Y_{i}-W_{ki}/W_{\text{max}}\right]\rangle }_{p(Y_{i}|Z_{k}=1)}\\ \Rightarrow \;W_{ki}=W_{\text{max}}\cdot {\langle Y_{i}\rangle }_{p(Y_{i}|Z_{k}=1)}\;. \end{array}$$

According to this analysis, the synaptic weight *W*_*ki*_ represents the expected value of input neuron *y*_*i*_ at the moment of a postsynaptic spike in network neuron *z*_*k*_ in a linear manner. In the compound memristive synapse, this expectation is encoded in the *M* + 1 possible weight states *W*_*ki*_ = 0, ω, …, *W*_max_ of the synapse. The linear encoding (8) is compatible with the convergence points which we observed previously in the small STDP pairing experiment in Figure 1F.

The above analysis only serves as an intuition and is no substitute for a thorough mathematical treatment of the learning process. A rigorous formal derivation that, for instance, also takes into account the dynamically changing response properties of the network neurons due to recurrent interactions and plastic changes in the weights, is provided in Methods. It reveals that the above intuition holds. More precisely, the likelihood distributions *p*(***Y*** | *Z*_*k*_ = 1) that are optimized in a WTA circuit with compound-synapse STDP are given by the product of the likelihoods of individual inputs

(9) $$p(Y=y(t)|Z_{k}=1)=\prod_{i=1}^{N}p(Y_{i}=y_{i}(t)|Z_{k}=1)\;,$$

and the likelihood for each individual input channel *y*_*i*_ is given by a Gaussian distribution

(10) $$p(Y_{i}=y_{i}(t)|Z_{k}=1)=\frac{1}{\sqrt{2\pi \sigma^{2}}}\cdot e^{-\frac{{\left(y_{i}(t)-\mu_{ki}\right)}^{2}}{2\sigma^{2}}}\;.$$

The mean values μ_*ki*_ and the standard deviation σ of the likelihood distributions (10) are identified as

(11) $$\mu_{ki}=W_{ki}/W_{\text{max}}=m_{ki}/M\text{ and }\sigma =1/\sqrt{(}W_{\max})\;.$$

Hence, the mean μ_*ki*_ of the distribution for input channel *y*_*i*_ is given by the fraction of active constituents in the compound memristive synapse. The width $\sigma =1/\sqrt{(}W_{\max})$ of the distribution is determined by the maximum weight *W*_max_ = *M* · ω and could, for instance, be controlled by the weight contribution ω of an individual stochastic switch. The resulting probabilistic model of the WTA network is a *Mixture-of-Gaussians* generative model (see Methods for a formal definition).

In order to illustrate the computational properties of this generative model, the likelihood distribution *p*(*Y*_*i*_ | *Z*_*k*_ = 1) for a single input *y*_*i*_ and a single active hidden cause *Z*_*k*_ = 1 is sketched in Figure 2B. An active hidden cause *Z*_*k*_ = 1 assigns probabilities to all possible instantiations *y*_*i*_(*t*) of *Y*_*i*_. In principle, the Gaussian likelihood distribution supports arbitrary real-valued input instantiations *Y*_*i*_(*t*) ∈ ℝ. We will come back to this observation in the Discussion section where we address possible extentions of the WTA network to support more complex input types. In this article, we consider only binary inputs that take on the value 0 (input pulse absent) or 1 (input pulse present), see the presynaptic pulses in Figure 1A. The corresponding likelihood values *p*(*Y*_*i*_ = 0 | *Z*_*k*_ = 1) and *p*(*Y*_*i*_ = 1 | *Z*_*k*_ = 1) are determined by the mean μ_*ki*_ and the variance σ of the likelihood distribution, see Equations 10 and (11). The task of the network when presented with an input ***y***(*t*) is to infer the posterior distribution over hidden causes *p*(*Z*_*k*_ = 1 | ***Y*** = ***y***(*t*)) and produce spikes according to this distribution. The optimal solution is given by Bayes rule (7) with the likelihood *p*(***Y*** = ***y***(*t*) | *Z*_*k*_ = 1) given by Equations (9) and (10), and an (input independent) a priori probability *p*(*Z*_*k*_ = 1). As we prove in Methods, the response distribution *p*_net_(***Z*** | ***Y*** = ***y***(*t*)) of the spiking WTA network implements a close and well-defined variational approximation of this posterior distribution. A minimal example of such Bayesian inference is sketched in Figure 2C, where we consider a small WTA network with only one input *y*_*i*_ and two network neurons *z*_*k*_ and *z*_*j*_. For a given input instantiation *y*_*i*_(*t*) the values *p*(*Y*_*i*_ = *y*_*i*_(*t*) | *Z*_*k*_ = 1) and *p*(*Y*_*i*_ = *y*_*i*_(*t*) | *Z*_*j*_ = 1) measure the likelihoods of the two competing hypotheses that neuron *z*_*k*_ or neuron *z*_*j*_ is the hidden cause of the observed input *y*_*i*_(*t*). These likelihood values shape the Bayesian posterior distribution (7) by contributing one factor to the product in Equation (9).

As a consequence, a network neuron *z*_*k*_ is particularly responsive to those input configurations ***y***(*t*) that are associated with high likelihood values *p*(***Y*** = ***y***(*t*) | *Z*_*k*_ = 1). The likelihood distributions are determined by the means μ_*ki*_, i.e., by the number *m*_*ki*_ of active switches in the synapses that change according to the compound-synapse STDP rule. Through this mechanism, synaptic plasticity governs the emergence of prototypic patterns that the network neurons are most responsive to, and thereby turns each neuron *z*_*k*_ into a probabilistic expert for certain input configurations ***y***(*t*). The aim of learning in the WTA network is to distribute the probabilistic experts *z*_*k*_ such that the likelihood of the presented input is (on average) as high as possible. This objective is an equivalent formulation of Maximum-Likelihood learning, and the *log-likelihood function*, which measures the average (logarithm of the) input likelihood, is a widely-used measure to determine how well a learning system is adapted to the presented input. Formally, the learning process can be described within the framework of generalized Expectation-Maximization (Dempster et al., 1977; Habenschuss et al., 2012; Nessler et al., 2013). In Methods we show that the generalized Expectation-Maximization algorithm is implemented in the WTA network via the interplay of the compound-synapse STDP rule (3), that adapts the synaptic weights *W*_*ki*_, and the homeostatic intrinsic plasticity rule, that regulates the intrinsic excitability *b*_*k*_ of the neurons such that each neuron maintains a long-term average firing rate. The interplay of synaptic and intrinsic plasticity achieves the aim of Maximum-Likelihood learning in the WTA network in the following sense:

The expected synaptic weight changes $\langle \frac{d}{dt}W_{ki}\rangle$ of the compound memristive synapses on average increase a lower bound of the log-likelihood function in a Mixture-of-Gaussians generative model during online learning until a (local) optimum is reached.

### 2.4. Demonstration of unsupervised learning

**Figure 3:**
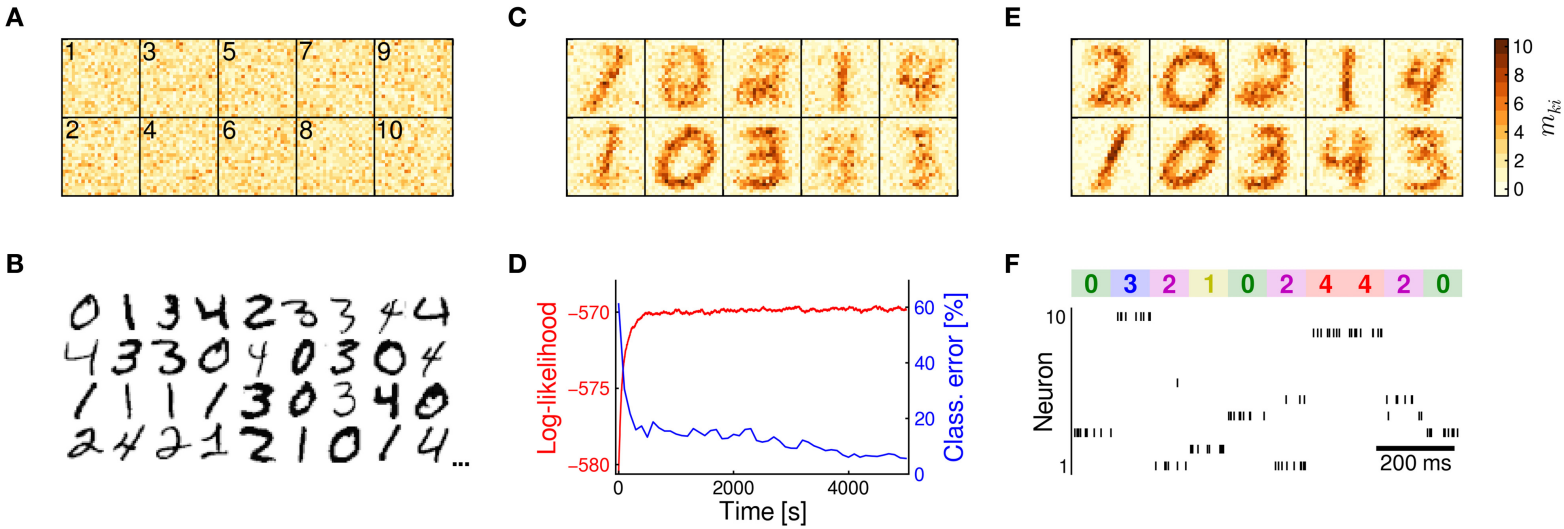
**Learning of hand-written digits**. **(A)** Synaptic weights at time *t* = 0 *s* after random initialization. Color intensities show the number *m*_*ki*_ ∈ [0, 10] of active switches for each connection. The indices *k* of the postsynaptic neurons are indicated in the top-left corners. **(B)** Examples from the MNIST data set. Pixel intensities of the digits were encoded as Poisson spike trains and presented to the network. Digits 0–4 were presented with a new digit being shown every 100 ms. **(C)** Weight matrix at time *t* = 200 s. Memristive synapses start to integrate salient features of the input stream. **(D)** Over the course of learning the log-likelihood function (red) increases, indicating that the network continuously refines its implicit statistical model of the presented data. This refinement leads to an improved classification performance (blue) on an independent test set. **(E)** At the end of the learning experiment at *t* = 5000 *s*, the synapses have specialized on different prototypes of the presented digits, rendering each network neuron a probabilistic expert for a certain digit. **(F)** Network response for 1 *s* at the end of learning. The presented digit is shown at the top. The input is transformed into a sparse and reliable spike code. Some digits invoke a spike response of more than one neuron. This ambiguous response encodes uncertainty in the variational posterior distribution.

We tested the learning capabilities of the compound memristive synapse model in a standard machine learning task for hand-written digit recognition. In a computer simulation, we set up a WTA network with *N* = 24 × 24 input neurons and *K* = 10 network neurons. Each synaptic weight *W*_*ki*_ was composed of *M* = 10 stochastic bistable switches, each contributing ω = 0.1 in its active state. The switching probabilities π_up_ = π_down_ = 0.001 were set to a quite low value. This corresponds to a long integration time for gradual memory formation in order to assess the general ability of the synapse model in online learning tasks. Before training, the stochastic switches were initialized randomly as shown in Figure 3A. The network was then exposed to hand-written digits 0–4 from the MNIST training data set (LeCun et al., 1998). Examples from the data set are shown in Figure 3B. Each pixel was encoded by one input neuron *y*_*i*_. Digits were presented as Poisson spike trains with firing rates depending on pixel intensities. Overall, the network was trained in an unsupervised setup for 5000 s with a new digit being presented every 100 ms. Figure 3C shows the weight matrix in an early stage of learning at *t* = 200 s. At this stage, the synapses begin to integrate salient statistical features of the input, such as the generally low activity along the frame. Furthermore, a specialization to certain digit classes becomes apparent for some of the network neurons.

Over the course of learning, the synapses continuously improve the network's implicit generative model of the presented input. This refinement is reflected in the log-likelihood function shown in Figure 3D that measures how well the probabilistic model is adapted to the input distribution. The ongoing refinement also becomes apparent by a more intuitive–and practically more relevant–measure, namely the classification performance of the network on an independent test set of hand-written digits. The classification error (blue in Figure 3D; see Methods for details) continuously decreases as training progresses. The improved performance on an independent test set furthermore indicates that the network develops a generally well-suited representation of the input and evades the risk of over-fitting.

At the end of training, after *t* = 5000 s, a set of prototypic digits has emerged in the compound memristive synapses as shown in Figure 3E. The well-adapted synapse array turns each network neuron into a probabilistic expert for a certain digit class. As a consequence, the network has learned to transform the *N*-dimensional, noisy spike input into a sparse and reliable spike code, as shown in Figure 3F. Typically, exactly one network neuron *z*_*k*_ fires in response to the input. But also the seemingly unclear cases, when two neurons respond simultaneously, carry meaningful information in a Bayesian interpretation: Since network spikes approximate the posterior distribution *p*(***Z*** | ***Y*** = ***y***(*t*)) through sampling, an ambiguous spike response encodes the level of uncertainty during probabilistic inference.

### 2.5. Influence of synaptic resolution

In the previous section, we have demonstated that the compound memristor synapse model is able to learn statistical regularities in the input stream and enables the spiking WTA network to perform probabilistic inference in a well-defined generative model. The demonstration employed compound synapses with *M* = 10 stochastic switches per synapse. Notably, the number of constituents *M* is a free parameter of the model and determines the weight resolution of the compound synapse. Increasing the synaptic resolution by recruiting more bistable switches per synapse is generally expected to improve the accuracy of the input representation, but comes at the cost of reduced integration density in a neuromorphic design. In the following, we therefore explore the opposite direction, i.e., unsupervised learning with a low weight resolution.

**Figure 4:**
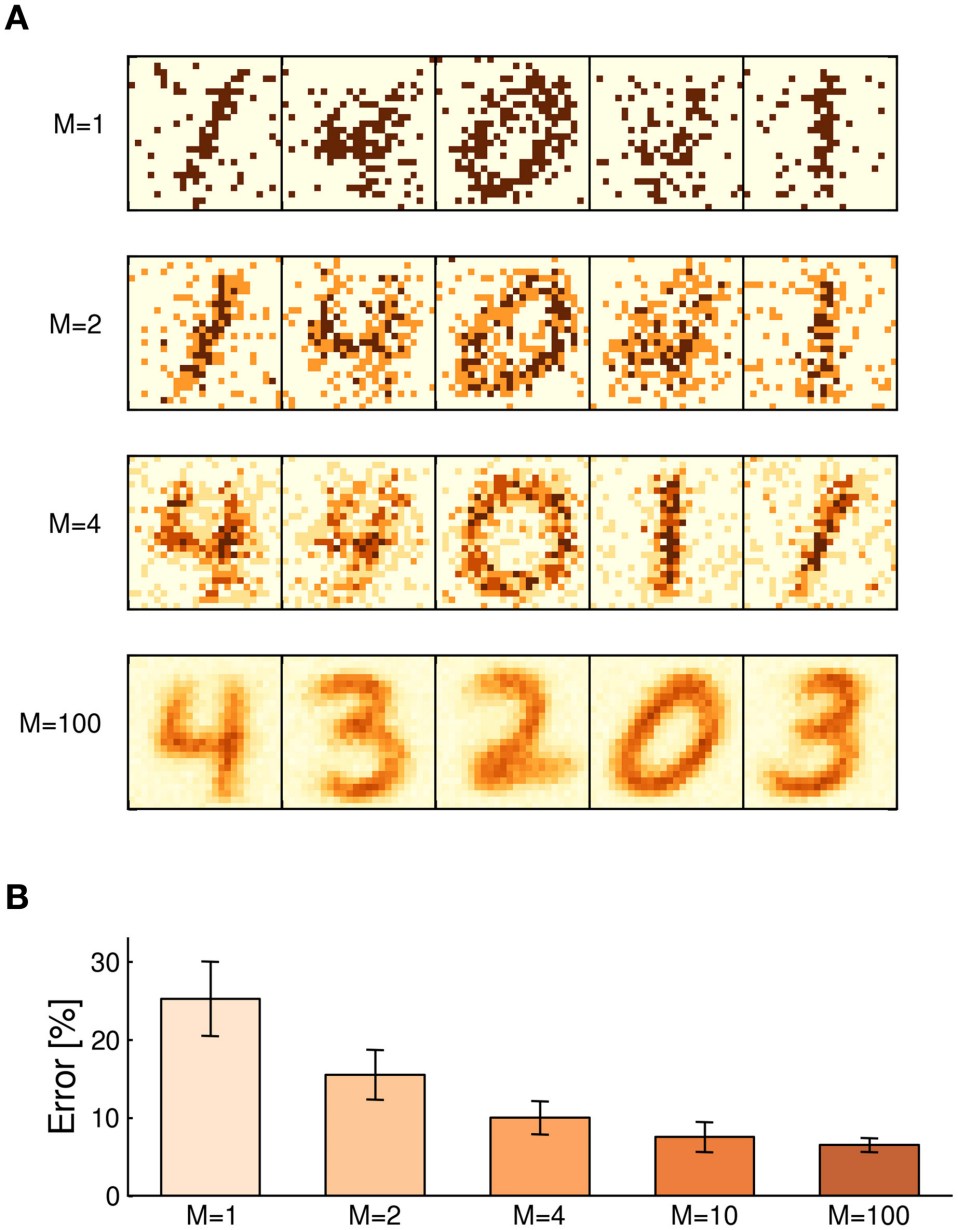
**Influence of the synaptic resolution**. **(A)** Examples of learned weight matrices with different numbers of constituents *M* per synapse. Even binary weights with only one stochastic switch learn to maintain a coarse image of prototypic digits. In the limit of large *M*, compound synapses support a quasi-continuous weight spectrum. **(B)** Classification error for different weight resolutions after 5000 s of learning, based on 20 independently trained networks for each value of *M*. Errorbars: *SD* among networks.

For estimating the influence of the weight resolution on the learning capabilities of the WTA network, we repeated the above computer simulation for different values of *M*, while holding the maximum weight *W*_max_ = ω · *M*, and thus the variance $\sigma =1/\sqrt{(}W_{\max})$ of the implicit generative model, fixed. Figure 4A shows examples of the digits stored in the synapse array after 5000 s of learning for *M* = 1, 2, 4, and 100 stochastic switches per synapse. Even binary synapses with *M* = 1 successfully identify noisy archetypes of the input digits. This observation is in line with previous studies on learning with binary weights (Fusi, 2002). The accuracy of the representation quickly increases with higher *M*-values. As an (academic) reference, we also included a simulation with *M* = 100 switches per synapse which support a quasi-continuous state spectrum.

The resulting ability of the WTA network to recognize hand-written digits is shown in Figure 4B in terms of the classification error on a test set. Each bar depicts the mean performance of 20 independently trained networks per *M*-value, errorbars show the standard deviation among networks. Taking the academic example with *M* = 100 as a reference for the performance achievable by the small WTA network, the computer simulations suggest that as few as *M* = 4 constituents per synapse may be sufficient for practical applications. While individual weights *W*_*ki*_ only store little information (ca. 2.3 bits in case of *M* = 4) about the expected input in channel *y*_*i*_, the partial evidence received from each of the *N* = 576 input channels is integrated by the network in a statistically correct manner to form a sharply peaked posterior, most of the time.

### 2.6. Robustness to device variations

We have demonstrated so far that spiking networks with compound memristive synapses can learn a faithful representation of their input when synapses consist of idealized bistable constituents. Large-scale physical implementations, however, are likely to exhibit substantial device variabilities and imperfections. Plasticity in the compound synapse model depends on two device properties that are likely to be distorted in physical implementations: the conductance of each individual constituent ω and the switching probabilities π_up_/π_down_. In the following, we address the impact of distortions in these two properties on the WTA network learning capabilities, separately.

We first turned to the conductance value ω of individual switches and investigated the robustness of learning to two fundamentally different types of noise in ω, namely spatial noise and temporal noise:

- *Spatial noise* describes device-to-device variations and addresses peculiarities of individual memristors that remain stable over time.
- *Temporal noise* refers to trial-to-trial variations and covers device instabilities over the course of learning.

**Figure 5:**
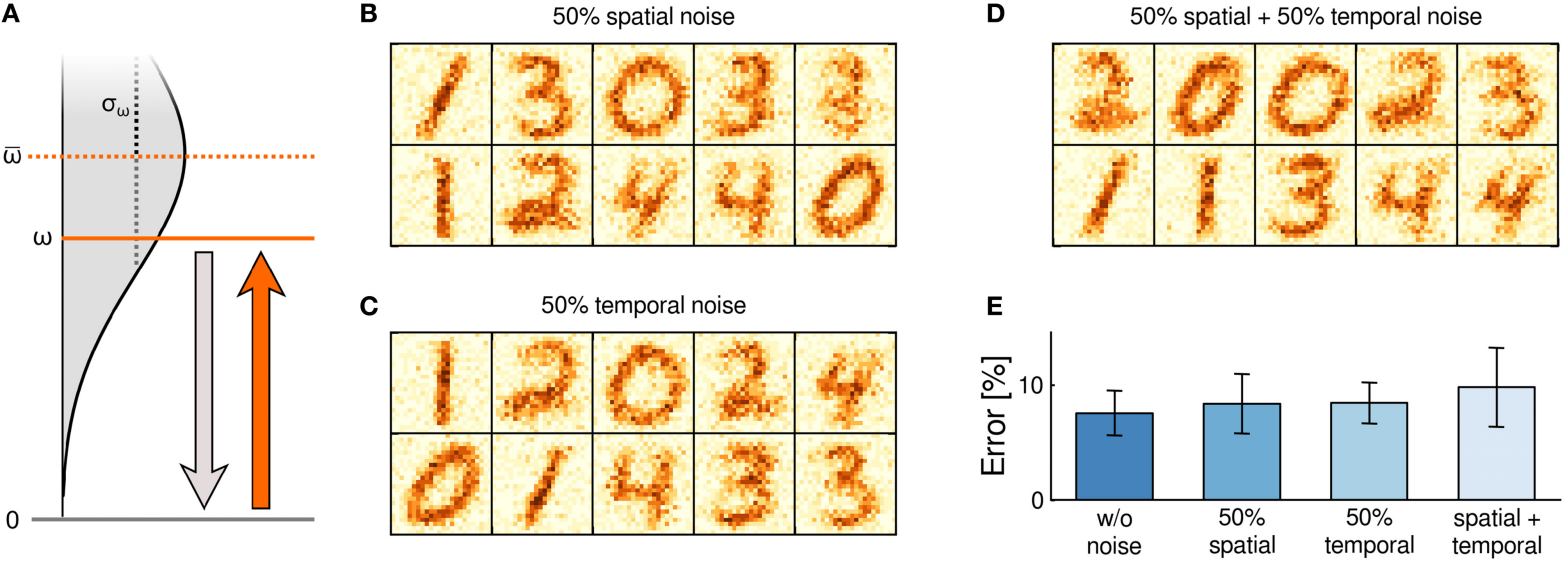
**Robustness to spatial and temporal noise**. **(A)** Noise was added by drawing the weight ω of the active state from a normal distribution. Three types of noise were tested: For spatial noise, a value ω ~ 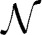
(ω; ω, σ^2^_ω_) was assigned to each switch and maintained throughout the experiment. For temporal noise, the value ω was redrawn after every LTP transition. For spatial + temporal noise, the device specific spatial weight value determined the mean for redrawing ω after LTP transitions. **(B–D)** Number *m*_*ki*_ of active switches after learning at *t* = 5000 s. Shown are example networks for all three noise types. **(E)** Classification error after learning, based on 20 independently trained networks per noise type. Errorbars denote standard deviation among networks.

Both types of noise can be suspected to induce serious disturbances during learning: In case of spatial noise, although device-to-device variations could average out if many memristors are employed, any remaining deviations give rise to sustained systematic errors that may build up over the course of learning. In case of temporal noise, while trial-to-trial variations could average out over time, any synaptic update rests upon a disturbed instantiation of the weight matrix, i.e., on a noisy (and false) assumption. In computer simulations, we accounted for these types of noise separately by disturbing the active-state weight value ω of the switches as sketched in Figure 5A. To model spatial noise, the weight ω was randomly drawn prior to training for each stochastic switch from a normal distribution 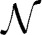
(ω; ω, σ^2^_ω_) with mean ω and standard deviation σ_ω_. To capture the effect of temporal noise, in contrast, the weight ω was redrawn from 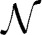
(ω; ω, σ^2^_ω_) whenever the constituent switched to its active state in an LTP transition. Furthermore, we examined the combined effect of both noise types being present simultaneously. In this combined case, the mean value for temporal noise was determined by the device-specific spatially perturbed weight value of each constituent. In any case, the range of perturbed weights was truncated to ω ≥ 0 to rule out negative conductances.

We repeated the experiment of Figure 3 under each of these noise conditions. The mean ω = 0.1 was set to the undisturbed weight value of the previous, idealized experiment. The noise level was set to σ_ω_ = 0.05, i.e., to 50% of the mean. Example cases of weight matrices after learning (shown are the *m*_*ki*_'s from individual simulation runs) are presented in Figures 5B–D for the three noise conditions “spatial,” “temporal” and “spatial+temporal,” respectively. Surprisingly, hardly any difference to the idealized setup is observable. Nevertheless, under 20 repetitions of the learning simulation the detrimental influence of noise becomes visible in the classification performance (see Figure 5E) as noise appears to slightly increase the mean of the classification error. In summary, these results reveal a remarkable robustness of learning with compound memristive synapses to substantial device variability and severe temporal instability.

We next turned to the question how distorted switching probabilities π_up_ and π_down_ influence the learning dynamics in the WTA network. In the theory section, we had assumed that π_up_ = π_down_, i.e., that the switching probabilities underlying LTP and LTD are balanced. This assumption, which could to some extent be achieved in a calibration step, yielded the elegant learning rule (3) and thereby facilitated the theoretical analysis. A physical implementation, however, will likely exhibit unbalanced switching probabilities in the majority of memristors. We examined the influence of unbalanced switching, in two ways. First, we applied spatial noise to the switching probabilities of individual constituents by drawing π_up_ and π_down_ (separately) from normal distributions with 50% noise level (truncated to 0 ≤ π_up_, π_down_ ≤ 1). Thus, about half of the stochastic switches were more responsive to LTP pulses, the other half more to LTD pulses; even more, some of the constituents only showed switching in one direction, or were completely unresponsive. Nevertheless, synapses developed a faithful representation of prototypic digits in a repetition of the experiment in Figure 3 (data not shown). Also the classification performance was only mildly impaired (*classification error*: 8.7 ± 2.7% based on 20 networks) compared to ideal, noisefree synapses (*classification error*: 7.5 ± 1.9%).

**Figure 6:**
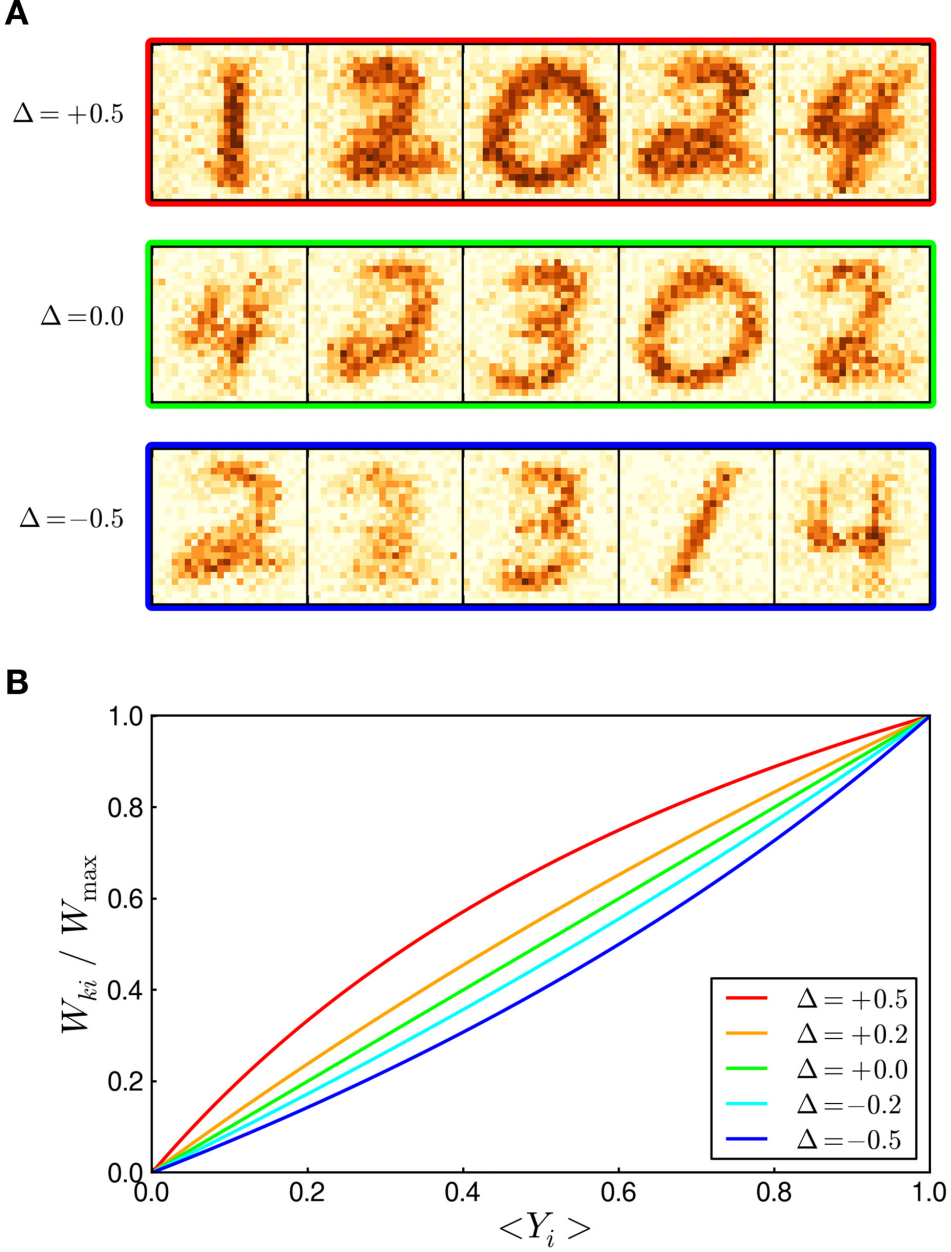
**Robustness to unbalanced switching probabilities**. **(A)** Examples of learned weight matrices when the switching probabilities π_up_ and π_down_ are systematically unbalanced. The parameter Δ: = (π_up_ − π_down_)/π_up_ measures the relative imbalance between LTP and LTD transitions. The top (bottom) row shows weight matrices resulting from a 50% decrease (increase) of the LTD switching probability. The balanced case is shown in the middle row for comparison. **(B)** Systematic imbalance (Δ ≠ 0) leads to changed equilibrium points in the STDP rule and can be theoretically understood as a non-linear encoding of the expected input by the synaptic weights.

In a second step, we pursued a slightly different–more principled–approach that permits a theoretical interpretation of how the altered synapse dynamics give rise to a different encoding of the expected input by the synaptic weights. Instead of drawing random parameters for each constituent, we systematically chose the LTD switching probability π_down_ larger (or smaller) than the LTP probability π_up_ throughout the synapse array. This systematic imbalance displays a worst-case scenario during learning since all synapses either favor (or suppress) LTD over LTP. We denote the relative imbalance between π_up_ and π_down_ by Δ: = (π_up_ − π_down_)/π_up_. For instance, Δ = −0.5 means that the probability for LTD transitions is 50% higher than for LTP transitions. Figure 6A shows examples of weight matrices after 5000 s of learning in a repetition of the experiment in Figure 3. In the top row, Δ = +0.5, LTP transitions are favored over LTD transitions, resulting in generally stronger weights in comparison with balanced STDP (Δ = 0.0, middle row). Conversely, in the bottom row, Δ = −0.5, the systematic strengthening of LTD leads to weaker weight patterns. Nevertheless, synaptic weight values converged to a dynamic equilibrium in either case since the STDP rule preserves its general stabilizing weight dependence. As can be expected from the prototypic digits that emerged in the weight matrices, the classification performance of the WTA networks was not considerably impaired by the unbalanced switching (classification errors estimated from 20 networks: 6.7 ± 0.8% for Δ = +0.5; 7.5 ± 1.9% for Δ = 0; 11.8 ± 4.0% for Δ = −0.5). Indeed, positive Δ-values even performed slightly (but not significantly) better than balanced switching. A conceptual understanding of the altered learning dynamics can be obtained from the equilibrium points of the unbalanced STDP rule. The short calculation, that had led to Equation (8) for the balanced case, can be repeated for unbalanced switching probabilities. Figure 6B shows the resulting encoding of the expected input value 〈*Y*_*i*_ 〉_*p*(*Y*_*i*_ | *Z*_*k*_ = 1)_ by the synaptic weight *W*_*ki*_ for different Δ-values. The example digits shown in panel A correspond to the red, green and blue graph in panel B, respectively. This analysis illustrates how unbalanced switching probabilities give rise to a non-linear encoding of the input in the WTA network. In particular, it can be seen how the same expected input value 〈*Y*_*i*_ 〉_*p*(*Y*_*i*_ | *Z*_*k*_ = 1)_ leads to stronger weights for Δ > 0, and weaker weights for Δ < 0.

## 3. Discussion

We have proposed the compound memristive synapse model for neuromorphic architectures that employs multiple memristors in parallel to form a plastic synapse. A fundamental property of the synapse model is that individual memristors exhibit stochastic switching between two stable memristive states rather than obeying a deterministic update rule. Yet, the expected weight change of the compound memristive synapse, as it arises from the stochastic switching of its constituents, yielded an STDP-type plasticity rule with a stabilizing, linear weight dependence. We examined the computational capabilities of the compound-synapse STDP rule in WTA networks, a common circuit motif in cortical and neuromorphic architectures, by analyzing the network and synapse dynamics from the perspective of probability theory and machine learning. The comprehensive mathematical treatment revealed that compound memristive synapses enable a spiking network to perform Bayesian inference in and autonomous statistical optimization of a Mixture-of-Gaussians generative model via generalized Expectation-Maximization. Accompanying computer simulations demonstrated the practical capability of the synapse model to perform unsupervised classification tasks and, furthermore, revealed a remarkable robustness of the compound synapses to substantial device variations and imperfections.

### 3.1. Comprehensive learning theory of memristive plasticity

Our work contributes a theoretical foundation for memristive learning in neural networks to the endeavor to employ memristors as plastic synapses in self-calibrating systems. Snider (2008); Querlioz et al. (2011), and Serrano-Gotarredona et al. (2013) have investigated how different pre- and postsynaptic waveforms can shape a memristive STDP learning window. Jo et al. (2010) and Mayr et al. (2012) have demonstrated STDP-type plasticity in Ag/Si and BiFeO3 memristors. Yu et al. (2013) reported stochastic switching between stable states in oxide-based memristive synapses. Gaba et al. (2013) studied the parameter dependence of switching probabilities in metal filament based memristors, indicating a renewal process that is independent of the overall network firing rate. A strategy for integrating nanoscale memristive synapses into a hybrid memristor-CMOS network architecture was proposed by Indiveri et al. (2013). The beneficial contribution of stochasticity to learning with CMOS synapse circuits was explored by Chicca et al. (2014). Here we have established a firm link between the emergent synapse configurations observed in such architectures (see e.g., Querlioz et al., 2011) and a rigorous mathematical description of memristive learning on the system level using machine learning theory. Our findings on memristive learning from a Bayesian perspective build upon a series of theoretical contributions on synaptic learning in spiking neural networks: Nessler et al. (2009, 2013) identified a general link between STDP-type synaptic plasticity and statistical model optimization for probabilistic inference in WTA networks. Habenschuss et al. (2012) extended this work to incorporate also homeostatic intrinsic plasticity, thereby overcoming several limiting assumptions on the input presentation. Finally, Habenschuss et al. (2013) investigated how the learning framework can be generalized to support a broad class of probability distributions. We expect that utilizing machine learning theory for describing the effects of specific memristor synapse models can significantly promote our understanding of memristive learning and its computational prospects.

### 3.2. Heading for a full hardware integration

Plasticity in the compound memristor synapse model relies on stochastic transitions between two stable states. Such bistable devices–or more generally, devices with a clearly discrete state spectrum–were reported to exhibit a high degree of uniformity (Lee et al., 2006a; Fang et al., 2011) and temporal stability (Indiveri et al., 2013). Notably, the theoretical approach we have persued in this work could likely be extended to cover memristors with more than two stable states and to support more complex input and plasticity mechanisms. For instance, the Gaussian likelihood distributions *p*(***Y*** | ***Z***) identified in the present study, in principle support inference over arbitrary real-valued input states ***y***(*t*). Such states could arise if the input is presented in the form of exponentially decaying or additive postsynaptic potentials. Such more complex input types could afford more versatile STDP pulsing schemes, and the resulting memristor plasticity rules could likely be incorporated in an adapted model of statistical learning. The reason we restricted the input to binary values *y*_*i*_(*t*) is found in the STDP pulsing scheme that employs binary presynaptic waveforms. In this case, the theoretically derived generative model reveals how active and inactive inputs contribute to the network's spike response by means of a Gaussian likelihood distribution *p*(*Y*_*i*_ = *y*_*i*_(*t*) | *Z*_*k*_ = 1) that is sampled only at *y*_*i*_(*t*) = 0 and *y*_*i*_(*t*) = 1.

In this article, we have employed a simple model for stochastic switching in memristive devices where switching occurs with probabilities π_up_, π_down_ which depend on the applied voltage difference across the memristor terminals. This phenomenological model captures the most salient aspects of switching in real memristive materials (Jo et al., 2009b; Gaba et al., 2013) and was used as an abstraction of memristive switching in a recent experimental study (Suri et al., 2013). In future research, it will be important to evaluate the effectiveness of this model either with physical memristors or in simulations based on detailed memristor models. The authors of Suri et al. (2013) raised the concern that the precise switching probabilities of individual devices are potentially hard to control in large-scale systems. In this regard, our simulation results indicate that learning with compound memristive synapses tolerates significant noise levels in the switching probabilities. We expect the origin for the observed robustness to be twofold: Firstly, imbalances in π_up_, π_down_ between different constituents are expected to partly average out in compound synapses according to the central limit theorem; secondly, the stabilizing weight dependence of compound-synapse STDP ensures that even unbalanced switching leads to stable weight configurations, albeit with slightly shifted convergence points.

Another potential issue for learning with compound memristive synapses is the absolute value of the switching probability. The product π_up_ · *W*_max_ can be linked to a learning rate in the theory domain (see Table 1) which controls how many samples from the input history are integrated into the implicit generative model during online learning. A slow and gradual memory formation, which is desirable for developing a representation of large and complex input data sets, relies on small learning rates, i.e., on small switching probabilities. It has to be seen if memristive materials that exhibit stochastic switching provide sufficiently small switching probabilities. A possible remedy in a hardware integration could be to multiplex the back-propagating signals from network neurons such that only a random subset of the memristors is notified of a network spike at a time (Fusi, 2002).

**Table 1:** **Correspondence of synapse parameters between the hardware and theory domain**.

**Parameter name**	**Hardware**	**Theory**
Learning rate	π_up_ · *W*_max_	η_*W*_
Max. weight *W*_max_	ω · *M*	1/σ^2^
Likelihood mean μ_*ki*_	*m*_*ki*_/M	σ^2^ · *W*_*ki*_
Synaptic resolution	ω	1/(*M* · σ^2^)

Regarding the physical model neurons of a hybrid memristor-CMOS architecture, two types of currents occur in the WTA network. The input integration via forward-synapses is spike based and could be realized with standard leaky integrators (see Methods). Lateral inhibition, in contrast, depends on the neuronal membrane potentials, and the involved inhibitory circuits should ideally transmit potentials instead of spikes. Alternatively, the effect of lateral inhibition could be approximated in a spike-based manner by populations of inhibitory neurons. Independent of the specific implementation of lateral inhibition, the resulting potential *u*_*k*_ − *u*_inh_ controls the stochastic response of the WTA neurons that could either be implemented genuinely with a stochastic firing mechanism or be emulated with integrate-and-fire neurons (Petrovici et al., 2013).

### 3.3. Inherently stochastic nature of compound-synapse STDP

The spiking WTA network architecture with compound memristive synapses exploits stochasticity in various ways, in that the stochastic firing of network neurons in response to a transient input trajectory triggers stochastic STDP updates in the synaptic weights. From a learning perspective, the high degree of stochasticity contributes to the network's ongoing exploration for potential improvements in the parameter space. While the learning theory only guarantees convergence to a local optimum of the weight configuration, the stochastic nature of the ongoing exploration enables the network to evade small local optima in the parameter landscape, and thereby improves the robustness of learning (compared to traditional batch Expectation-Maximization).

For the derivation of the learning algorithm, we have focused on the weight-dependent STDP rule (3) which describes the expected temporal weight change $\langle \frac{d}{dt}W_{ki}\rangle$ of the compound synapse. The stochasticity of memristive switching, however, gives rise to a probability distribution over the weights, as well. Indeed, in equilibrium we expect that the number of active constituents *m*_*ki*_ follows a binomial-type weight distribution. This points to a potential knob for adjusting the amount of stochasticity used during online learning: when many memristors are recruited per synapse, i.e., for large *M*, we expect a reduced variance in the weight distribution.

Besides the level of stochasticity, the parameter *M* also controls the weight resolution of the compound synapse. In Figure 4, we have investigated the impact of the weight resolution on the learning capabilities of the WTA network. Notably, we observed that even with *M* = 4, i.e., with synapses that feature only 5 weight levels, the network performed reasonably well in the hand-written digit recognition task. The observation that even a low synaptic weight resolution can yield a satisfactory performance has important practical implications for nanoscale circuit designs, where integration density and power consumption impose crucial constraints, since the area allocated by the synapse array grows linearly in the synaptic size *M*. For instance, SRAM cells can be fabricated with a cell size of 0.127μm^2^ (Wu et al., 2009), corresponding to a memory density of ≲ 1 Gb/cm^2^. Importantly, this estimate does not include any additional plasticity circuits for implementing STDP or similar plasticity mechanisms. Functional Ag/Si memristive crossbars with 2 Gb/cm^2^ memory density were demonstrated by Jo et al. (2009a), with densities up to 10 Gb/cm^2^ being envisioned (Jo et al., 2009a,b). In the long term, memristive crossbars are expected to combine the advantages of SRAM and Flash memory regarding energy efficiency, non-volatility and integration density (Yang et al., 2013).

### 3.4. Generalization to other materials and future research

In recent years, a plethora of (in a broader sense) memristive materials has been discovered, and the characterization and refinement of their switching dynamics is evolving rapidly. At least four types of stochastically switching memristive devices can be distinguished: Switching in (1) anion-based (e.g., HfO_x_/TiO_x_ Yu et al., 2013) and (2) cation-based (e.g., Ag/GeS_2_ Suri et al., 2013) devices mainly originates from conductive filament formation (Yang et al., 2013). In contrast, (3) single-electron latching switches [e.g., CMOS/MOLecular (CMOL) CrossNets Lee et al., 2006b] rely on electronic tunneling effects and, thus, their stochastic switching dynamics arise directly from the underlying physical process. Similarly, (4) magnetoresistive devices (e.g., spin-transfer torque magnetic memory (STT-MRAM) Vincent et al., 2014) can inherit stochastic switching dynamics from fundamental physical properties. Some manufacturing processes related to these ideas (like conductive-bridging RAM and STT-MRAM) reached already an early industrial stage, others are still primarily subject of academic research. While the microscopic origins of plasticity in these memristor types are fundamentally different, they all share stochastic, persistent switching between bistable memory states on a phenomenological level. We therefore believe that the compound memristive synapse model displays a promising concept for future work in diverse research fields.

Independent of the underlying switching mechanism, any nanoscale synaptic crossbar will likely exhibit imperfections and imbalances due to process variations. Here, we have investigated spatial and temporal noise in the weight values as well as deviations in the switching probabilities under unbiased, uniform conditions. However, physical implementations can be expected to also suffer from more systematic imperfections, such as structural imbalances (e.g., one corner of the array being more reactive) or crosstalk between neighboring devices. While our computer simulations indicate a remarkable general robustness against device variations of various types, additional research is required to estimate the influence of such systematic, and potentially coupled, deviations.

### 3.5. Conclusions

In this article, we have introduced the compound memristive synapse model together with the compound-synapse STDP rule for weight adaptation. Compound-synapse STDP, a stabilizing weight-dependent plasticity rule, naturally emerges under a standard STDP pulsing scheme. In addition, by employing memristors with bistable memristive states, compound memristive synapses may circumvent practical challenges in the design of reliable nanoscale memristive materials. Both, our theoretical analysis and our computer simulations confirmed that compound-synapse STDP endows networks of spiking neurons with powerful learning capabilities. Hence, the compound memristive synapse model may provide a synaptic design principle for future neuromorphic hardware architectures.

## 4. Methods

### 4.1. Probabilistic model definition

The probabilistic model that corresponds to the spiking network is a mixture model with *K* mixture components and Gaussian likelihood function. Formally, we define a joint distribution *p*(***Y*** = ***y***(*t*), ***Z*** = ***z***(*t*) | **θ**) over *K* hidden binary random variables ***Z*** = (*Z*_1_, …, *Z*_*K*_)^T^ with values *z*_*k*_(*t*) ∈ {0, 1}, and *N* real-valued visible random variables ***Y*** = (*Y*_1_, …, *Y*_*N*_)^T^ with values *y*_*i*_(*t*) ∈ ℝ. The parameter set **θ** = {**$\hat{b}$**, ***W***} consists of a real-valued bias vector **$\hat{b}$** = ($\hat{b}$_1_, …, $\hat{b}$_*K*_)^T^ and a real-valued *K* × *N* weight matrix ***W***. The hidden RVs *Z*_*k*_ display an unrolled representation of a multinomial RV ˜ *Z* ∈ {1, …, *K*} that enumerates the mixture components, and we identify $\tilde{Z}$ = *k* ⇔ *Z*_*k*_ = 1, i.e., exactly one binary RV *Z*_*k*_ is active in the random vector ***Z***. In the following, we stick to the unrolled vector notation ***Z***, and, for readability, omit the time-dependent notation and further shorten the notation by identifying the RVs with their values, e.g., we write *p*(***z*** | **θ**) for *p*(***Z*** = ***z***(*t*) | **θ**).

The likelihood distribution fulfills the naïve Bayes property, i.e., all statistical dependencies between visible RVs *y*_*i*_, *y*_*j*_ are explained by the hidden state ***z***. More precisely, the generative model has the following structure:

(12) $$p(y,z|\theta )=p(z|\theta )\cdot \prod_{k=1}^{K}\prod_{i=1}^{N}p{\left(y_{i}|z_{k}=1,\theta \right)}^{z_{k}}\;,$$

with the prior

(13) $$p(z|\theta )=\frac{e^{z^{T}\hat{b}}}{\sum_{j=1}^{K}e^{{\hat{b}}_{j}}}$$

and the likelihood

(14) $$p\left(y_{i}|z_{k}=1,\theta \right)=\frac{1}{\sqrt{2\pi \sigma^{2}}}\cdot e^{-\frac{{\left(y_{i}-\mu_{ki}\right)}^{2}}{2\sigma^{2}}},$$

with σ^2^ denoting an arbitrary (but fixed) variance which displays a constant in the model. Equation (14) defines a Gaussian likelihood model for each input RV *Y*_*i*_ with mean μ_*ki*_ which is selected by the active hidden cause *z*_*k*_ = 1 in Equation (12). For theoretical considerations, it is convenient to reorganize Equation (14) according to its dependency structure:

(15) $$p\left(y_{i}|z_{k}=1,\theta \right)=\frac{e^{-y_{i}^{2}/(2\sigma^{2})}}{\sqrt{2\pi \sigma^{2}}}\cdot e^{\frac{\mu_{ki}}{\sigma^{2}}\cdot y_{i}}\cdot e^{-\mu_{ki}^{2}/(2\sigma^{2})}$$

(16) $$\;=:h(y_{i})\cdot e^{W_{ki}\cdot y_{i}}\cdot e^{-A_{ki}},$$

where we set *W*_*ki*_ = μ_*ki*_/σ^2^ and *A*_*ki*_ = μ^2^_*ki*_/(2 σ^2^) = σ^2^ *W*^2^_*ki*_/ 2. The first factor does neither depend on the hidden causes *z*_*k*_ nor on the weight *W*_*ki*_ and will play no role during inference and learning. The second factor describes the coupling between the visible RV *y*_*i*_ and the active latent RV *z*_*k*_ through the mean *W*_*ki*_ = μ_*ki*_/σ^2^. Finally, the third factor solely depends on the weight *W*_*ki*_ (and not on *y*_*i*_) and ensures correct normalization of the distribution.

### 4.2. Inference

The posterior distribution given an observation ***Y*** follows directly from Bayes rule:

(17) p(z|y,θ)=p(z|θ)· p(y|z,θ)/Norm.

(18) $$\begin{array}{l} =e^{z^{T}\hat{b}}\prod_{k=1}^{K}\prod_{i=1}^{N}h{\left(y_{i}\right)}^{z_{k}}\cdot \\ \;e^{z_{k}\cdot W_{ki}\cdot y_{i}}\cdot e^{-z_{k}\cdot A_{ki}}/\text{Norm.} \end{array}$$

(19) $$=e^{z^{T}\cdot \left[\hat{b}-A+W\cdot y\right]}\cdot \prod_{i=1}^{N}h(y_{i})/\text{Norm.}$$

with ***A***: = (*A*_1_, …, *A*_*k*_, …, *A*_*K*_)^T^ and $A_{k}:=\sum_{i\;=\;1}^{N}A_{ki}$. We evaluate the posterior for a specific hidden RV *z*_*k*_ to be active and provide the normalization constant explicitly:

(20) $$p\left(z_{k}=1|y,\theta \right)=\frac{e^{{\hat{b}}_{k}-A_{k}+\sum_{i=1}^{N}W_{ki}\cdot y_{i}}\cdot \prod_{i=1}^{N}h(y_{i})}{\sum_{j=1}^{K}e^{{\hat{b}}_{j}-A_{j}+\sum_{i=1}^{N}W_{ji}\cdot y_{i}}\cdot \prod_{i=1}^{N}h(y_{i})}$$

(21) $$\;=\frac{e^{{\hat{u}}_{k}}}{\sum_{j=1}^{K}e^{{\hat{u}}_{j}}}$$

where we defined ${\hat{u}}_{k}={\hat{b}}_{k}-A_{k}+\sum_{i\;=\;1}^{N}W_{ki}\;\cdot \;y_{i}$. The quantities *û*_*k*_ are reminiscent of neuronal membrane potentials which consist of bias terms $\hat{b}$_*k*_ − *A*_*k*_ and synaptic input $\sum_{i\;=\;1}^{N}W_{ki}\;Y_{i}$. However, implicitly the bias terms depend on all afferent synaptic weights since ${\hat{b}}_{k}-A_{k}={\hat{b}}_{k}-\frac{\sigma^{2}}{2}\sum_{i}W_{ki}^{2}$ and, thus, rely on information not locally available to the neurons. This issue will be resolved in the context of learning: We will identify update rules for both biases and synapses which only use information available locally and thereby make a neural network implementation feasible.

### 4.3. Learning via generalized expectation-maximization

We investigate unsupervised learning of the probabilistic model based on generalized online Expectation-Maximization (EM) (Dempster et al., 1977), an optimization algorithm from machine learning theory. To this end, we impose additional constraints on the posterior distribution (Graça et al., 2007) which will enable a neural network implementation via homeostatic intrinsic plasticity (Habenschuss et al., 2012) and STDP-type synaptic plasticity (Habenschuss et al., 2013; Nessler et al., 2013). Since the derivation is almost identical to Habenschuss et al. (2012), we only outline the key steps and main results in the following and refer to Habenschuss et al. (2012) for a the details.

The algorithmic approach rests upon the generalized EM decomposition:

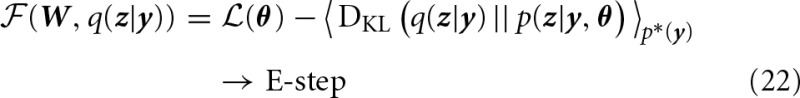



(23) $$\begin{array}{ll} \begin{array}{l} ={\langle \log p(y,z|\theta )\rangle }_{p^{*}(y)q(z|y)}+{\langle H(q(z|y))\rangle }_{p^{*}(y)}\\ \;\;\;\;\;\to \text{M}-\text{step} \end{array} & \;(23) \end{array}$$

with the log-likelihood 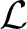
(**θ**) = 〈log *p*(***y*** | **θ**)〉_p^*^(***y***)_, the Kullback-Leibler divergence D_KL_(*q*(***z***)‖*p*(***z***)) = ∑_***z***_ *q*(***z***) · log (*q*(***z***)/*p*(***z***)) and the entropy *H*(*q*(***z***)) = − ∑_***z***_ *q*(***z***) · log *q*(***z***). The distribution p^*^(***y***) denotes the input distribution actually presented to the system. The distribution *p*(· | **θ**) is the probabilistic model defined above. The distribution *q*(***z***|***y***) is called variational posterior and will ultimately be implemented by the spiking network. The short hand notation 〈·〉_p^*^(***y***) *q*(***z***|***y***)_ denotes the concatenated average 〈〈·〉_*q*(***z***|***y***)_ 〉_p^*^(***y***)_ with respect to the input distribution and the resulting variational posterior. In principle, the above decomposition holds for any choice of *q*, and since the Kullback-Leibler divergence in Equation (22) is strictly non-negative, the objective function 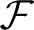 is a lower bound of the log-likelihood 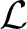. During optimization the algorithm will persue two goals: to increase 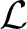, i.e., to better adapt the probabilistic model to the data, and to keep 〈D_KL_(*q* ‖ *p*)〉 small, i.e., to maintain a reliable approximation *q*(***z*** | ***y***) of the exact posterior *p*(***z*** | ***y***, **θ**).

We first impose a homeostatic constraint on the variational posterior *q*(***z***|***y***), namely that the long term average activation of any hidden RV *z*_*k*_ matches a predefined target value *c*_*k*_ (with ∑_*k*_ *c*_*k*_ = 1). Formally, we define a set of constrained distributions 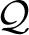 = {*q*: 〈*z*_*k*_ 〉_p^*^(***y***) *q*(***z***|***y***)_ = *c*_*k*_ ∀ 1 ≤ *k* ≤ *K*} and demand *q*(***z***|***y***) ∈ 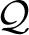. The optimization algorithm then relies on the joint application of an E(expectation)-step and an M(aximization)-step: During the E-step, we aim to minimize the Kullback-Leibler divergence with respect to *q* ∈ 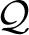 in Equation (22); during the M-step, we perform gradient ascent on 〈log *p*(***y***, ***z*** | **θ**)〉 with respect to the weights *W*_*ki*_ in Equation (23). The E- and M-step will be discussed separately.

The E-step is a constrained optimization problem, namely the minimization of 〈D_KL_(*q* ‖ *p*)〉 such that *q* ∈ 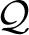, that can be solved through Lagrange multipliers. Since we imposed *K* constraints (one per RV *z*_*k*_), we need *K* Lagrange multipliers β_*k*_. It turns out that the solution to this optimization problem simply adds the multipliers β_*k*_ to the biases $\hat{b}$_*k*_ − *A*_*k*_ in Equation (20). This convenient result gives rise to the definition of intrinsic excitabilities *b*_*k*_: = $\hat{b}$_*k*_ − *A*_*k*_ + β_*k*_ which unify biases and multipliers in a single quantity. Furthermore, it turns out that the optimal values of the β_*k*_'s (and thus the *b*_*k*_'s) can be determined via iterative update rules that solely rely on the hidden RVs *z*_*k*_ under the variational response *q*(***z*** | ***Y***) and overwrite the non-local terms *A*_*k*_. In summary, we obtain the variational posterior distribution *q* that solves the E-step:

(24) $$q(z|y)=\frac{e^{u_{k}}}{\sum_{j=1}^{K}e^{u_{j}}}\text{ with }u_{k}=b_{k}+\sum_{i=1}^{N}W_{ki}\cdot y_{i}$$

(25) $$\Delta b_{k}\propto \;{\langle c_{k}-z_{k}\rangle }_{p^{*}(y)q(z|y)}\;.$$

The variational posterior in Equation (24) is described in terms of membrane potentials *u*_*k*_ which consist of synaptic input ∑_*i*_ *W*_*ki*_ *Y*_*i*_ and intrinsic excitabilities *b*_*k*_. Equation (25) regulates the intrinsic excitabilities *b*_*k*_ in a homeostatic fashion: When the average response exceeds the target *c*_*k*_, the excitability is reduced, and vice versa.

The M-step can be solved via gradient ascent on 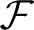 with respect to the weights *W*_*ki*_. The variational posterior *q* is a constant during the M-step in EM, and thus the log-joint distribution log *p*(***y***, ***z*** | **θ**) remains as the only *W*_*ki*_-dependent term in Equation (23). By taking the derivative of the log-joint defined by Equation (12), (13), and (16) with respect to *W*_*ki*_, we obtain:

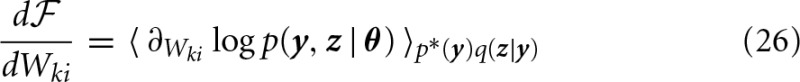



(27) $$=\;{\langle \partial_{W_{ki}}z_{k}\cdot \log p\left(y_{i}|z_{k}=1,\theta \right)\rangle }_{p^{*}(y)q(z|y)}$$

(28) $$=\;{\langle z_{k}\cdot (y_{i}-\sigma^{2}\cdot W_{ki})\rangle }_{p^{*}(y)q(z|y)}\;.$$

The gradient with respect to the weights *W*_*ki*_ yields Hebbian-type update rules that use pre-(*y*_*i*_) and post-(*z*_*k*_) synaptic activity and the current weight *W*_*ki*_ given the input p^*^(***y***) and the variational response *q*(***z***|***Y***). Importantly, only local information is required during the E- and M-step.

### 4.4. Spiking network implementation

The spiking neural network model instantiates Equation (24), (25), and (28), i.e., it represents the variational posterior *q*(***z*** | ***y***) for probabilistic inference through its spike response and implements the derived update rules for generalized online EM learning through intrinsic and synaptic plasticity.

Each of the RVs *z*_*k*_ is represented by one of the *K* network neurons, and each spike in the network is a sample from the variational posterior *q*(***z*** | ***y***) by identifying *z*_*k*_ = 1 for a spike of the k-th network neuron. By setting the instantaneous firing rate ρ_*k*_ to be

(29) $$\rho_{k}=\underset{\delta t\to 0}{\lim}p(\text{spike in}[t,t+\delta t])/\delta t=r_{\text{net}}\cdot e^{u_{k}-u_{\text{inh}}}$$

with $u_{\text{inh}}:=\log\sum_{j\;=\;1}^{K}\exp(u_{j})$ the network thus implements Equation (24) for any choice of *r*_net_, i.e., *p*_net_ = *q*.

The learning rules (25) and (28) rely on expected values 〈·〉_p^*^(***y***) *q*(***z***|***y***)_. The expectations can be approximated from input samples ***y*** ~ p^*^(***y***) and posterior samples ***z*** ~ *q*(***z***|***y***) in response to this input. The input vector ***y*** is defined at any time *t* in the network as it measures the instantaneous presence or absence of rectangular input pulses. Samples of the latent variable ***z***, in contrast, are only defined at the spike times of the network. Hence integrating expected values 〈*z*_*k*_〉 from the spike response can be expressed most conveniently in terms of the spike train function *s*_*k*_(*t*) = ∑_*f*_ δ(*t* − *t*^*f*^_*k*_) of the network neurons. We obtain the following plasticity rules:

(30) $$\frac{db_{k}}{dt}=\eta_{b}\cdot \left[r_{\text{net}}\cdot c_{k}-s_{k}(t)\right]$$

(31) $$\frac{dW_{ki}}{dt}=\eta_{W}\cdot s_{k}(t)\cdot \left[y_{i}-\sigma^{2}\cdot W_{ki}\right]$$

with small learning rates η_*b*_ and η_*W*_. The homeostatic rule (30) regulates the intrinsic excitabilities *b*_*k*_ such that the average target activations 〈*z*_*k*_(*t*)〉 ≈ *c*_*k*_ are maintained over the presentation of many different input patterns ***y***(*t*) ~ p^*^(***y***) in accordance with Equation (25), and thereby implements the E-step. Building on the network response shaped by the E-step, the synaptic rule (31) on average increases the objective function 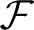 since synaptic changes $\frac{d}{dt}W_{ki}$ on average point in the direction of the ***W***-gradient of 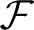 given by Equation (28), thereby implementing the M-step. Since synaptic updates rely on a (sufficiently) precise E-step which, in turn, needs to integrate any changes in the network response due to synaptic plasticity, homeostatic intrinsic plasticity is required to act on faster time scales than synaptic plasticity. As a consequence, the learning rate η_*b*_ will typically exceed the learning rate η_*W*_ in the spiking network implementation.

The homeostatic intrinsic plasticity rule (30) can readily be implemented by the spiking neurons: The intrinsic excitability *b*_*k*_ of each neuron increases linearly in time with a slow drift η_*b*_ · *r*_net_ · *c*_*k*_ and is lowered abruptly by η_*b*_ at the spike times of neuron *z*_*k*_. Similarly, mapping the synaptic plasticity rule (31) to the compound-synapse STDP rule (3) is straight-forward due to the structural equivalence of Equations (3) and (31): The learning rate η_*W*_ in the theory domain corresponds to π_up_ · *W*_max_ in the hardware domain, e.g., high jumping probabilities π_up_ and large weight contributions ω = *W*_max_/*M* of individual stochastic switches lead to high learning rates η_*W*_. Furthermore, the maximum weight *W*_max_ can directly be identified with the precision 1/σ^2^ of the likelihood distribution (14). In the theory domain, we know that μ_*ki*_ = σ^2^ · *W*_*ki*_, and hence, μ_*ki*_ = *W*_*ki*_/*W*_max_ = *m*_*ki*_/*M* for the compound synapses. Finally, due to the structural equivalence of Equations (3) and (31) we find that the compound memristor plasticity rule (3) inherits the convergence properties from the theoretically derived plasticity rule (31) during online learning. The resulting translation of memristor synapse parameters to the abstract model is summarized in Table 1.

### 4.5. Computer simulations

All computer simulations were performed with customized Python scripts. For the computer simulations, we employed a network architecture with *K* = 10 network neurons and *N* = 24 · 24 = 576 inputs. The simulation time step was δ *t* = 1 ms and the PSP time constant τ = 10 ms. The overall network firing rate was set to *r*_net_ = 100 Hz, the homeostatic target activation uniformly to *c*_*k*_ = 1/K. Synapses were composed of *M* = 10 constituents with weight ω = 0.1 each. Switching probabilities were set to π_up_ = π_down_ = 10^−3^. This corresponds to a Gaussian likelihood model with variance σ^2^ = 1 and learning rate η_*W*_ = 10^−3^. The learning rate for homeostatic intrinsic plasticity was set to η_*b*_ = 20 · η_*W*_. For the simulations in Figures 4–6, certain parameters deviated from the above, depending on the simulation setup. For Figure 6, the switching probability π_up_ = 10^−3^ was kept fixed, and π_down_ was adapted for different Δ-values. All other changes are described directly in the Results section.

For learning experiments, digits 0, 1, 2, 3, 4 were extracted in equal proportion from the MNIST training data set (LeCun et al., 1998). A frame of two pixels width was removed, leaving images of size 24 × 24. The images (indexed by *s*) were scaled linearly to activity patterns *x*^*s*^_*i*_ ∈ [0.05, 0.9], with *i* = 1, …, *N*, which were presented to the network as follows. For given activity pattern **x**^*s*^ = (*x*^*s*^_1_, …, *x*^*s*^_*N*_), each input *i* spiked with probability *p*^*spike*^_*i*_ = 1 − (1 − *x*^*s*^_*i*_)^δ *t*/τ^ per time step δ*t*. During training, a new activity pattern ***x***^*s*^ was randomly drawn from the training set every 100 ms. Each network was trained for 5000 s. To obtain the unweighted PSP values *y*_*i*_, the resulting spike patterns were convolved with a box kernel of duration τ and amplitude 1, and then clipped to values [0, 1]. This defined the input ***y***(*t*), and thus (implicitly) the data distribution ***y***(*t*) ~ p^*^(***y***). Notably, the spiking probability *p*^spike^_*i*_ is chosen such that 〈*y*_*i*_〉 = *x*^*s*^_*i*_.

The estimate of the log-likelihood in Figure 3D was based on 5000 input samples ***y***(*t*), which were randomly drawn from the training data, and assumed a uniform prior *p*(*z*_*k*_ = 1 | **θ**) = 1/*K* in accordance with the homeostatic target activation. The classification performance in Figures 3D, 4B, 5E was determined as follows. For given configuration of the synapses and intrinsic excitabilities, 100 versions of each digit from the training data set were presented to the network for 1 s each. Each neuron was labeled to be tuned to the digit class it was most responsive to. Then 500 versions of each digit from the MNIST test data set were presented to the network for 1 s each. The network neuron that spiked most during the 1 s period determined the network's classification of the input digit. The classification error is the fraction of wrongly classified digits.

### 4.6. Implementation with leaky integrator neurons

The idealized stochastic neurons in the WTA network model feature abstact membrane potentials *u*_*k*_ that integrate the input ***y***(*t*) through the weights *W*_*ki*_ linearly. In a hardware integration, however, synaptic weights arise from the conductance of memristors, and neurons are physical implementations based on capacitors and various other circuit elements. Here, we outline one possible hardware integration and consider a leaky integrator with membrane potential *U*_*k*_ that obeys the following dynamics:

(32) $$\tau_{\text{m}}\cdot \;\frac{dU_{k}}{dt}=-(U_{k}-B_{k})+I_{k}/G_{\text{L}}\;,$$

with membrane time constant τ_m_, leak conductance *G*_L_, resting potential *B*_*k*_ and synaptic input current *I*_*k*_. In the setup of Figure 1A, input spikes trigger a rectangular voltage pulse of duration τ and with amplitude *U*_pre_. Denoting the conductance of the memristive synapse by *G*, this generates a synaptic current *I* = *U*_pre_ · *G*. The equilibrium membrane potential (i.e., $\frac{d}{dt}U_{k}=0$) under this current is *U*_*k*_ = *B*_*k*_ + (*U*_pre_/*G*_*L*_) · *G*. For small membrane time constant τ_*m*_ → 0, e.g., for a small neuron capacitance, the fast membrane will closely resemble the rectangular presynaptic pulse shape, and the PSP amplitude (*U*_*k*_ − *B*_*k*_) will be proportional to the weight *G*. This linear integration property of *U*_*k*_ also holds in case of multiple memristive synapses acting in parallel, and we find

(33) $$U_{k}=B_{k}+\frac{U_{\text{pre}}}{G_{\text{L}}}\cdot \sum_{i}G_{ki}\cdot y_{i}\;.$$

Consequently, the membrane potential *U*_*k*_ of the leaky integrator matches the idealized membrane potential *u*_*k*_ employed in the Results section up to a linear function that serves to translate the voltage-based potential *U*_*k*_ to the unitless potential *u*_*k*_. Using the membrane potential *U*_*k*_, the exponential firing behavior (29) of the neurons could either be realized with an inherently stochastic firing mechanism. Alternatively, deterministic leaky integrate-and-fire neurons could be operated in a stochastic regime by adapting, for instance, the approach taken in Petrovici et al. (2013).

## Author contributions

Johannes Bill and Robert Legenstein conceived the study, designed the experiments and wrote the manuscript. Johannes Bill developed the theory and performed the computer simulations.

## Funding

Written under partial support of CHIST-ERA ERA-Net (Project FWF #I753-N23, PNEUMA) (Johannes Bill, Robert Legenstein) and the European Union project #604102 The Human Brain Project (HBP) (Robert Legenstein).

### Conflict of interest statement

The authors declare that the research was conducted in the absence of any commercial or financial relationships that could be construed as a potential conflict of interest.
